# NODULE INCEPTION Directly Targets *NF-Y* Subunit Genes to Regulate Essential Processes of Root Nodule Development in *Lotus japonicus*

**DOI:** 10.1371/journal.pgen.1003352

**Published:** 2013-03-21

**Authors:** Takashi Soyano, Hiroshi Kouchi, Atsuko Hirota, Makoto Hayashi

**Affiliations:** National Institute of Agrobiological Sciences, Tsukuba, Ibaraki, Japan; Virginia Tech, United States of America

## Abstract

The interactions of legumes with symbiotic nitrogen-fixing bacteria cause the formation of specialized lateral root organs called root nodules. It has been postulated that this root nodule symbiosis system has recruited factors that act in early signaling pathways (common *SYM* genes) partly from the ancestral mycorrhizal symbiosis. However, the origins of factors needed for root nodule organogenesis are largely unknown. *NODULE INCEPTION* (*NIN*) is a nodulation-specific gene that encodes a putative transcription factor and acts downstream of the common *SYM* genes. Here, we identified two Nuclear Factor-Y (NF-Y) subunit genes, *LjNF-YA1* and *LjNF-YB1*, as transcriptional targets of NIN in *Lotus japonicus*. These genes are expressed in root nodule primordia and their translational products interact in plant cells, indicating that they form an NF-Y complex in root nodule primordia. The knockdown of *LjNF-YA1* inhibited root nodule organogenesis, as did the loss of function of *NIN*. Furthermore, we found that *NIN* overexpression induced root nodule primordium-like structures that originated from cortical cells in the absence of bacterial symbionts. Thus, NIN is a crucial factor responsible for initiating nodulation-specific symbiotic processes. In addition, ectopic expression of either *NIN* or the *NF-Y* subunit genes caused abnormal cell division during lateral root development. This indicated that the *Lotus* NF-Y subunits can function to stimulate cell division. Thus, transcriptional regulation by NIN, including the activation of the *NF-Y* subunit genes, induces cortical cell division, which is an initial step in root nodule organogenesis. Unlike the legume-specific NIN protein, NF-Y is a major CCAAT box binding protein complex that is widespread among eukaryotes. We propose that the evolution of root nodules in legume plants was associated with changes in the function of NIN. NIN has acquired functions that allow it to divert pathways involved in the regulation of cell division to root nodule organogenesis.

## Author Summary

Legumes produce nodules in roots as the endosymbiotic organs for nitrogen-fixing bacteria, collectively called rhizobia. The symbiotic relationship enables legumes to survive on soil with poor nitrogen sources. The rhizobial infection triggers cell division in the cortex to generate root nodule primordia. The root nodule symbiosis has been thought to be recruited factors for the early signaling pathway from the ancestral mycorrhizal symbiosis, which usually does not accompany the root nodule formation. However, how the root nodule symbiosis-specific pathway inputs nodulation signals to molecular networks, by which cortical cell division is initiated, has not yet been elucidated. We found that NIN, a nodulation specific factor, induced cortical cell division without the rhizobial infection. NIN acted as a transcriptional activator and targeted two genes that encode different subunits of a NF-Y CCAAT box binding protein complex, *LjNF-YA1* and *LjNF-YB1*. Inhibition of the LjNF-YA1 function prevented root nodule formation. Ectopic expression of the NF-Y subunit genes enhanced cell division in lateral root primordia that is not related to root nodule organogenesis. The NF-Y genes are thought to regulate cell division downstream of NIN. NF-Y is a general factor widespread in eukaryotes. We propose that NIN is a mediator between nodulation-specific signals and general regulatory mechanisms associated with cell proliferation.

## Introduction

The interactions of legume plants with their bacterial symbionts, collectively called “rhizobia,” cause the formation of specialized root lateral organs called root nodules. Unlike lateral roots, of which initiation is regulated by endogenous signals, activation of the mitotic cell cycle for root nodule organogenesis is triggered by symbiont-derived signaling molecules called nodulation (Nod) factors [1]–[7]. Legumes have developed unique molecular networks that transmit exogenous signals to the regulatory factors for organ development. Over the past decade, many factors involved in nodulation processes have been identified, leading to basic models for the nodulation signaling pathways. However, the mechanisms by which nodulation signals induce cell division have not been elucidated.

Nodulation processes are initiated by the adhesion of rhizobia to root hairs in the model legume plants *Lotus japonicus* and *Medicago truncatula* [8]. Symbiotic bacteria are entrapped by curled root hairs and form microcolonies on the host epidermal cells. Subsequently, the invasion of plant tissues by the bacterial symbionts is mediated by host cell-derived tubular structures called infection threads. The infection threads develop by invagination of the plasma membrane at the infection foci, where the plant cell wall is degraded. Concomitant with the progression of infection processes at the epidermis, a fraction of cortical cells beneath the site of infection begin to divide and form a root nodule primordium.

Forward genetics studies in the two model legume plants have revealed that early signaling pathway(s) downstream of Nod factor perception are common to those required for mycorrhizal symbiosis. Genes involved in both symbiosis systems are referred to as the common *SYM* genes [9]. It has been postulated that the root nodule symbiosis systems have recruited functions partly from the ancestral mycorrhizal symbiosis systems. Among the proteins encoded by the common *SYM* genes, CCaMK (calcium/calmodulin-dependent protein kinase) plays a pivotal role in both symbioses. This protein is thought to act as a decoder of perinuclear Ca^2+^ oscillations triggered by Nod factor perception [7]. Gain-of-function (gof)-CCaMK rescues nodulation- and mycorrhizal infection-defective phenotypes caused by mutations in common *SYM* genes that are required for the Ca^2+^ oscillation [10], [11]. Furthermore, gof-CCaMK induces spontaneous root nodules in the absence of rhizobia [12], [13], indicating that this protein bypasses an early nodulation signaling pathway from Nod factor perception. The common *SYM* pathway is thought to transmit nodulation signals to pathway(s) that have evolved specifically to regulate root nodule organogenesis. In so doing it diverts the plant's general gene expression networks to execute complex nodulation processes.

In the current model, CCaMK-induced root nodule organogenesis is mediated by a cytokinin receptor called *Lotus* histidine kinase 1 (LHK1) [10], [11], [14], [15]. Cytokinin is a phytohormone that regulates various aspects of plant development [16]. Loss-of-function mutants of *LHK1* (*hit1*) and its *M. truncatula* counterpart, *CYTOKININ RESPONSE1* (*MtCRE1*), fail to initiate timely cortical cell division in response to rhizobial signals [15], [17]. Gof-LHK1 causes the development of spontaneous root nodules in loss-of-function *ccamk* mutants without rhizobial infection [10], [11], [14]. Exogenously applied cytokinin also causes the formation of spontaneous root nodules [18]. These results implicate cytokinin as an endogenous molecular signal for initiating root nodule organogenesis, and this signal must necessarily be integrated with nodulation specific pathways. *NODULATION SIGNALING PATHWAY1* (*NSP1*), *NSP2*, and *NODULE INCEPTION* (*NIN*) are required for CCaMK- and LHK1- mediated root nodule organogenesis [10], [11]. Unlike *LHK1* and *MtCRE1*, these genes are essential for both the rhizobial infection processes in the epidermis and the cortical responses [19], [20], [21]. They are thought to function in nodulation-specific processes [20], [22], [23], although recent findings have implied that NSP1 and NSP2 also act in mycorrhizal symbiosis [24], [25]. *NSP1* and *NSP2* encode GRAS family transcription factors [26], [27]. *NIN* encodes a putative transcription factor with a RWP-RK domain [19]. The absence of *NSP1*, *NSP2*, or *NIN* function results in abortion of spontaneous root nodule formation by gof-CCaMK and gof-LHK1 [10], [11]. Transcriptional regulation by these factors is important for root nodule organogenesis. However, the mechanisms by which these factors mediate rhizobial infection signals to activate developmental programs underlying nodulation have not yet been elucidated.

*NIN* was the first gene to be genetically characterized for its role in the regulation of nodulation processes [19]. This gene is involved in multiple processes including symbiotic root hair responses and infection thread formation in the epidermis, and induction of cell division in the cortex [19], [28]. *NIN* is expressed during the early stages of organogenesis in root nodule primordia generated by exogenously applied cytokinin [18]. Cytokinin-induced *NIN* expression is downregulated by mutations in *LHK1* and *MtCRE1* [15], [17]. On the other hand, the expression of both *NSP1* and *NSP2* is less sensitive to or downregulated by exogenous cytokinin [17], [29], although expression of these genes is required for *NIN* expression induced by rhizobial infection [23], [28]. The two GRAS transcription factors form a heterodimer and bind to the *M. truncatula NIN* promoter *in vitro* [30]. The expression pattern of *NIN* implies that this gene may be a primary regulator of cortical cell division in response to cytokinin signaling. Despite the functional importance of *NIN* in root nodule-specific symbiotic events, our knowledge of its function is at the genetic level. The precise function of NIN in nodulation, and the molecular properties of the protein, have not yet been fully elucidated. The identification of genes that regulate cortical cell division downstream of NIN will be important for understanding the molecular mechanisms underlying root nodule organogenesis.

In this study we biochemically and biologically dissected NIN protein function. We found that NIN acts as a transcriptional activator that induces cortical cell division in the absence of bacterial symbionts. Furthermore, we determined that genes encoding different subunits of Nuclear Factor Y (NF-Y) are direct targets of NIN. NF-Y is a heterotrimeric CCAAT box binding protein complex composed of subunits A, B, and C [31]. The genes that we identified as NIN targets encode subunits A and B. We named these genes *LjNF-YA1* and *LjNF-YB1*. The product of *LjNF-YA1* is orthologous to *M. truncatula* HAP2-1, which is involved in meristem persistence in indeterminate-type root nodules [32]. Our functional analyses indicate that the *NF-Y* genes play overlapping roles with that of *NIN* in root nodule organogenesis. Unlike the legume-specific NIN protein, NF-Y is a general factor widespread among eukaryotes. Ectopic expression of *NIN* and the *NF-Y* subunit genes also influenced cell division in lateral root primordia that is not related to root nodule organogenesis. We propose that NIN is a mediator between rhizobial infection signals and general regulatory mechanisms associated with cell proliferation.

## Results

### Identification of candidate NIN target genes

*NIN* encodes a protein containing a RWP-RK domain, which is conserved among all plants from algae to seed plants [33]. Predictions of the secondary structure of this domain suggest that it binds to DNA [19]. Xie et al showed that NIN binds to the promoter of the *L. japonicus nodulation pectate lyase* gene [34]. Therefore, NIN is predicted to be a transcription factor. Supporting this idea, a NIN-GFP fusion protein that was functional in *L. japonicus* root epidermal responses (Figure S1C) localized to nuclei in *Nicotiana benthamiana* leaves (Figure S1A, S1B), as did the Arabidopsis NIN-like protein 7 [35]. In addition, the NIN protein fused to the GAL4 DNA-binding domain induced expression of a *GFP-GUS* reporter that had four tandem repeats of a GAL4 target nucleotide sequence in its promoter (Figure S1D–S1F) [36]. These results suggest that NIN is a positive regulator of gene expression.

To search for candidate genes that are targeted by NIN, we took advantage of the publicly available transcriptome database (http://cgi-www.cs.au.dk/cgi-compbio/Niels/index.cgi) [37] and selected 19 genes as possible candidates (Figure S2A). The NIN-dependent expression of 9 candidates was examined and validated by real time (RT)-PCR (Figure S2B).

We performed knockdown analyses of several of these candidate NIN targets to investigate their functions in nodulation processes, and found that RNA interference (RNAi) of chr5.CM0571.340.r2.m inhibited root nodule formation (see below). chr5.CM0571.340.r2.m encodes NF-YA, which forms a heterotrimeric CCAAT box-binding protein complex with other two subunits, NF-YB and NF-YC. We further found the *NF-YB* gene (LjSGA_022269.1) among the other candidate NIN targets (Figure S2A). NF-Y subunits are required for nodulation processes in *M. truncatula* [32] and *Phaseolus vulgaris* [38]. In particular, *MtHAP2-1*, which is orthologous to chr5.CM0571.340.r2.m (Figure S3), is involved in the maintenance of meristem activity in indeterminate-type root nodules [32]. Furthermore, mammalian NF-Y also functions in the regulation of cell division [39]–[42]. We expected that the *Lotus* NF-Y subunits would be included in the same NF-Y complex, and that this complex might function in nodulation processes downstream of NIN. Therefore, we focused on the *Lotus NF-Y* subunit genes for further analyses, and named them *LjNF-YA1* (chr5.CM0571.340.r2.m) and *LjNF-YB1* (LjSGA_022269.1).

### *LjNF-YA1* and *LjNF-YB1* are direct transcriptional targets of NIN

**Figure 1:**
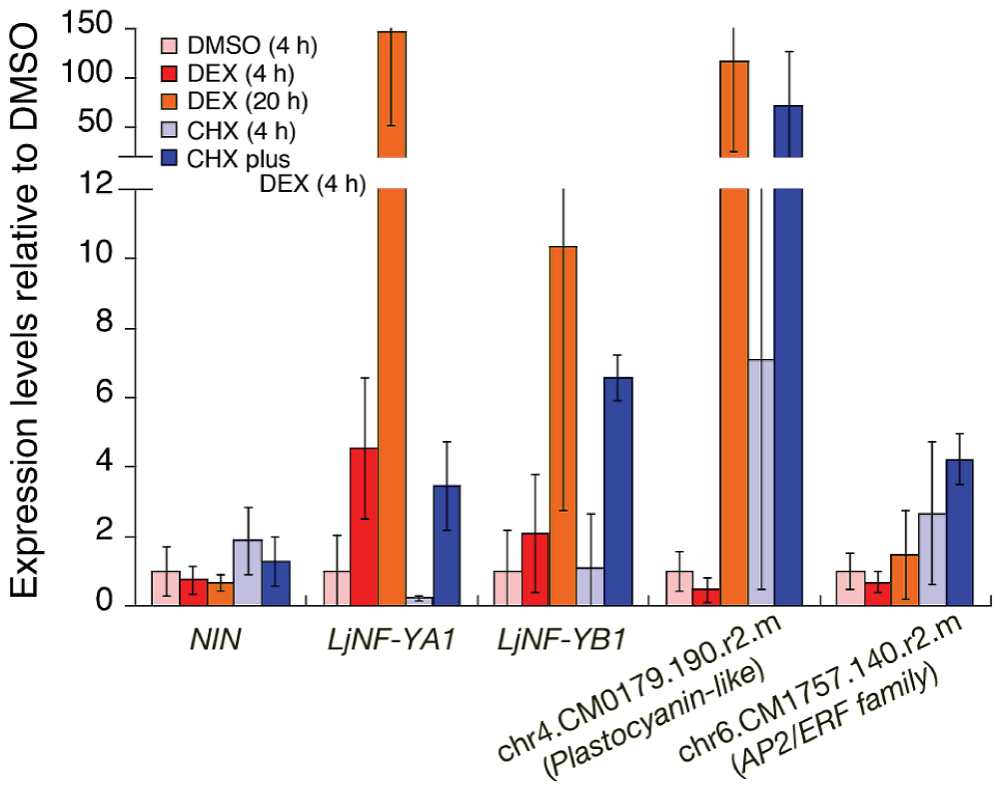
Expression of candidate NIN-target genes in roots that were ectopically expressing the NIN protein. RT-PCR was used to analyze gene expression in *nin-2* roots that were transformed with *Pro35S-NIN-GR*. Roots were treated as indicated in the figure. For CHX plus DEX, the DEX was added after pre-incubation with CHX for 30 min. chr4.CM0179.190.r2.m encodes a plastocyanin-like domain-containing protein (PLDP) and chr6.CM1757.140.r2.m encodes an AP2/ERF family protein. These genes are listed in Figure S2. The promoter of the former gene possesses NIN-binding nucleotide sequences (see Figure 2). The latter gene was used as a negative control for DEX treatment. The means and SDs from 3 biological repeats are shown.

To examine whether *NIN* overexpression induces expression of *LjNF-YA1* and *LjNF-YB1*, we expressed a NIN-GR fusion protein, in which the glucocorticoid receptor (GR) was fused to the C-terminus of NIN, in *nin-2* mutant roots. The overexpression construct was driven by the *CaMV35S* promoter (*Pro35S*). The transgenic roots were treated with or without dexamethasone (DEX) and cycloheximide (CHX). This recombinant protein is functional in *Lotus* roots, because when the gene construct was driven by the *NIN* promoter in the presence of DEX, it suppressed the infection thread-defective *nin-2* phenotype (Figure S4A and S4B). RT-PCR analysis showed that DEX treatment of roots transformed with the overexpression construct induced expression of *LjNF-YA1* and *LjNF-YB1* within 4 h, and that further incubation (20 h) resulted in increased levels of expression (Figure 1). Therefore, NIN induces expression of these genes. CHX treatment did not repress the expression induced by DEX. In the cases of *LjNF-YB1* and another candidate NIN target, chr4.CM0179.190.r2.m, which encodes a plastocyanin-like domain-containing protein (PLDP; see Figure S2A), CHX treatment enhanced the expression induced by DEX (Figure 1). These results support the idea that *LjNF-YA1* and *LjNF-YB1* are primary targets of NIN.

**Figure 2:**
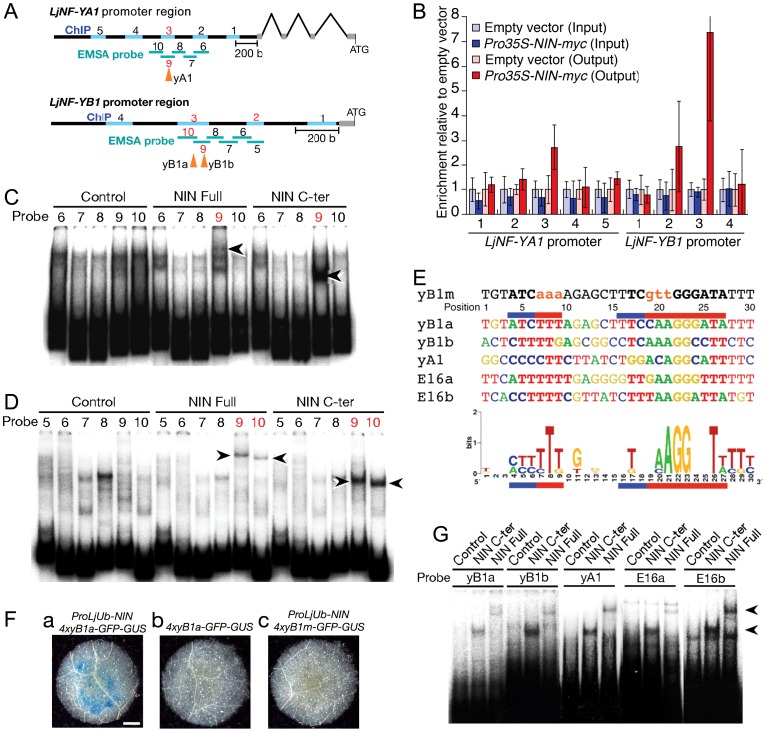
NIN directly targets *LjNF-YA1* and *LjNF-YB1*. (A) A diagram of the *LjNF-YA1* and *LjNF-YB1* promoter regions. These regions were used for the promoter-GUS reporters (see Figure 3). Gray lines indicate 5′-UTRs. Regions analyzed by RT-PCR for the ChIP assays in (B) are shown as blue lines and probes used for EMSA in (C) and (D) are shown as green lines. The red numbers indicate regions and probes that gave positive results in the ChIP assays and EMSAs. Arrowheads indicate positions of the NIN-binding sites. (B) ChIP assays using either *ProLjUb-NIN-my*c roots or control (empty vector) roots. The means and SDs from 3 biological repeats are shown. (C,D) EMSAs for analyzing NIN binding to *LjNF-YA1* (C) and *LjNF-YB1* (D) promoter regions. NIN-myc (Full), NIN(520–878)-myc (C-ter), and *in vitro* translation products without templates (control) were incubated with ^32^P-labeled probes shown in (A). Arrowheads indicate mobility-shifted bands specifically detected when incubated with NIN proteins. (E) An alignment of the partial nucleotide sequences of probes that were bound by NIN. yB1a, yB1b, yA1, E16a, are E16b correspond to NBSs found in the promoters of *LjNF-YB1*, *LjNF-YA1*, and the PLDP-encoding gene. Red and blue lines indicate nucleotides that are required for NIN-binding to NBS-yB1a, or that influence binding, respectively (see Figure S5). NBS-yB1m is an NBS-yB1a derivative with nucleotide substitutions. Comparison of NIN-binding sequences illustrated by logo is shown at the bottom. (F) GUS expression in tobacco leaf disks transformed with the indicated constructs. Bar: 1 mm. (G) EMSA for analyzing NIN binding to NBS-yB1a and the NBS-yB1a-like sequences. Arrowheads indicate probes that were bound by NIN proteins.

**Figure 3:**
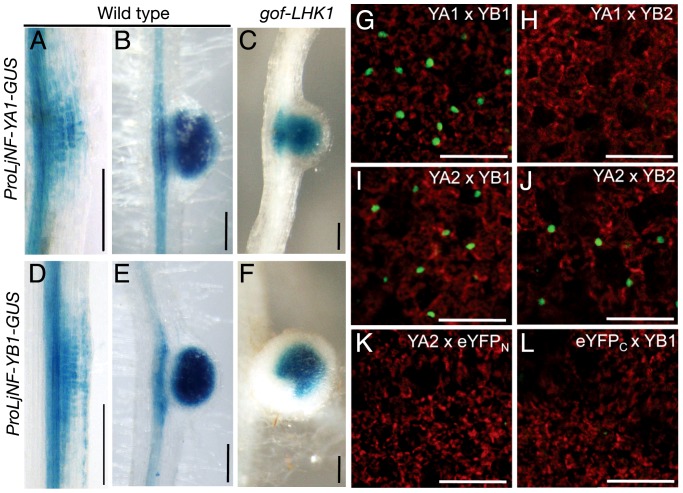
Spatial expression patterns of *LjNF-YA1* and *LjNF-YB1* during root nodule organogenesis and their protein interactions *in planta*. (A–F) GUS expression in roots that were transformed with either *ProLjNF-YA1-GUS* (A–C) or *ProLjNF-YB1-GUS* (D–F). GUS staining was detected in root nodule primordia (A, D), developing root nodules (B, E), and in spontaneous root nodules caused by *Pro35S-gof-LHK1* (C, F). (G–L) BiFC analysis of interactions between LjNF-YA and LjNF-YB subunits in *N. benthamiana* leaves. Confocal images of eYFP fluorescence (green) from nuclei and chloroplast autofluorescence (red) are shown. *ProLjUb-eYFP_C_-LjNF-YA1* was co-introduced with either *ProLjUb-eYFP_N_-LjNF-YB1* (G) or *ProLjUb-eYFP_N_-LjNF-YB2* (H). *ProLjUb-eYFP_C_-LjNF-YA2* was co-introduced with either *ProLjUb-eYFP_N_-LjNF-YB1* (I), *ProLjUb-eYFP_N_-LjNF-YB2* (J), or *ProLjUb-eYFP_N_* (K). *ProLjUb-eYFP_N_-LjNF-YB1* was co-introduced with *ProLjUb-eYFP_C_* (L). Bars: 0.2 mm in (A–F); 50 µm in (G–L).

We performed a chromatin immunoprecipitation (ChIP) analysis to investigate NIN binding to the *LjNF-YA1* and *LjNF-YB1* promoters *in vivo*. Chromatin suspensions were prepared from roots that were transformed with either *ProLjUb-NIN-myc* or an empty vector. The NIN-myc protein was expressed from the *L. japonicus polyubiquitin* promoter (*ProLjUb*) [43]. The recombinant NIN protein suppressed the infection thread-defective *nin-2* phenotype when expressed using the *NIN* promoter, indicating that this fusion protein is functional *in planta* (Figure S4C). Several primer sets for detecting *LjNF-YA1* and *LjNF-YB1* promoter fragments (indicated by blue lines in Figure 2A) were designed to cover the whole promoter regions that correspond to those used for spatial expression analyses using GUS reporter constructs (see Figure 3). Using anti-myc antibodies we detected enrichment of *LjNF-YA1* promoter region 3 and *LjNF-YB1* promoter regions 2 and 3 in immunoprecipitates of chromatin suspensions derived from roots expressing *ProLjUb-NIN-myc* (Figure 2A, 2B). The level of enrichment of *LjNF-YB1* promoter region 3 was approximately three times greater than those of *LjNF-YA1* promoter region 3 and *LjNF-YB1* promoter region 2. These results indicate that NIN binds to these promoter regions *in vivo*.

We also tested for *in vitro* binding of NIN to these promoter regions using electrophoresis mobility shift assays (EMSAs) with the NIN-myc protein and a NIN(520–878)-myc protein. The latter protein is the C-terminal half of NIN that contains the RWP-RK domain and the PB1 domain [19], [33]. Shifted bands were detected when *LjNF-YA1* probe 9 and *LjNF-YB1* probes 9 and 10 were incubated with the NIN proteins (Figure 2C, 2D). Differences in the mobilities between NIN-myc and NIN(520–878)-myc indicated that these mobility shifts were due to binding of the NIN proteins. These results show that NIN interacts with the *LjNF-YA1* and *LjNF-YB1* promoter regions that were enriched in the ChIP analysis, probably through the RWP-RK domain.

### Identification of NIN-binding nucleotide sequences

We performed detailed EMSAs to identify the DNA sequences bound by NIN in the *LjNF-YB1* promoter. An *LjNF-YB1* promoter region corresponding to probes 9 and 10 was divided into 6 shorter sequences that were used as probes (Figure S5Aa; probes 11–16). NIN(520–878)-myc bound only to probe 14 (Figure S5B). Assays using five derivatives of probe 14, each with a six-nucleotide substitution (see Figure S5Ab; m1 to m5), narrowed down the NIN-binding site to a sequence of 31 bp covered by the mutations in m3 to m5 (Figure S5C). Further analyses using probes with three-nucleotide substitutions revealed that two separate parts of the 31 bp sequence were required for NIN binding (Figure 2E; Figure S5Ab, S5D). We refer to this NIN-binding nucleotide sequence (NBS) as NBS-yB1a. To examine whether NBS-yB1a confers NIN-dependent expression on a *GFP-GUS* reporter gene, a fragment with four tandem repeats of NBS-yB1a was inserted upstream of a *CaMV35S* minimal promoter (*4xyB1a-GFP-GUS*). GUS staining was detected in tobacco leaves when the reporter construct was co-introduced with *ProLjUb-NIN* (Figure 2Fa,b). On the other hand, a similar construct with mutations in the NBS (*4xyB1m-GFP-GUS*; see Figure 2E) did not show GFP-GUS expression even if *ProLjUb-NIN* was co-introduced (Figure 2Fc). These results indicate that 4 tandem repeats of NBS-yB1a are sufficient for NIN-dependent gene expression.

We then searched for additional DNA sequences similar to NBS-yB1a, and found two NBS-yB1a-like sequences, referred to as NBS-yA1 and -yB1b, respectively, in the *LjNF-YA1* and *LjNF-YB1* promoters (Figure 2E). These sequences were included in the regions that were bound by the NIN protein (Figure 2A). NBS-yB1b overlaps with the region covered by probes 11, 12, and 13 in the *LjNF-YB1* promoter (Figure S5Ab). NBS-yB1a, NBS-yB1b, and NBS-yA1 are located 755, 712, and 1725 bp upstream of their respective putative translation initiation codons. We also found two NBS-yB1a-like sequences (NBS-E16a and -E16b) in the promoter of the PLDP-encoding gene that was also identified as a NIN target (Figure 2E). These sequences are located 363 and 193 bp upstream of the putative translation initiation codon. This PLDP-encoding gene was induced by the NIN-GR protein in the presence of CHX, as were *LjNF-YA1* and *LjNF-YB1* (Figure 1; Figure S2). ChIP analysis showed that NIN bound to the promoter of the PLDP-encoding gene *in vivo* (Figure S6). On the other hand, NBS-yB1a-like sequences were not found within 3 kb of the putative translation initiation codon of chr6.CM1757.140.r2.m, which encodes an AP2/ERF family protein. Expression of this gene was not induced by ectopic *NIN* expression (Figure 1). EMSAs showed that NIN proteins bound to probes containing the identified NBSs *in vitro* (Figure 2G). The specificity of NIN binding to these NBSs was confirmed using competition analyses (Figure S5E). These results are in agreement with the idea that NIN directly targets the promoters of *LjNF-YA1*, *LjNF-YB1*, and the PLDP-encoding gene and activates their transcription. A consensus of the NBSs identified here is shown in Figure 2E. A region on the left (positions 4–9) and one on the right (positions 16–27) of the consensus sequence corresponded to those required for NIN binding in NBS-yB1a. The left region was rich in T and C and was less conserved than the right region. The right region contained AGG at positions 21–23 and T at position 26, and these were present in all the NBSs that we identified in this study.

### *LjNF-YA1* and *LjNF-YB1* are co-expressed in the root nodule primordium

We used GUS reporter constructs to investigate the expression patterns of *LjNF-YA1* and *LjNF-YB1* during root nodule organogenesis. The promoter regions of *LjNF-YA1* and *LjNF-YB1* (2.8 and 1.5 kb upstream from the putative translation initiation codon, respectively; also see Figure 2A) were inserted upstream of the *GUS* reporter gene. These constructs were introduced into *L. japonicus* roots by *Agrobacterium rhizogenes*-mediated transformation. Histochemical GUS analysis revealed that *ProLjNF-YA1-GUS* and *ProLjNF-YB1-GUS* were expressed in the dividing cortical cells of early root nodule primordia (Figure 3A and 3D) and in developing root nodules (Figure 3B and 3E). Both genes were also expressed in root nodules that spontaneously developed in gof-LHK1 plants [10] in the absence of *Mesorhizobium loti* (Figure 3C and 3F). These results suggest that expression of *LjNF-YA1* and *LjNF-YB1* in root nodule primordia is regulated by LHK1-mediated networks. The expression patterns were similar to that of GUS expression from the *NIN* promoter [13]. A construct containing 4 repeats of NBS-yB1a (*4xyB1a-GFP-GUS*) was also expressed in *L. japonicus* root nodule primordia, whereas a mutated version of the construct (*4xyB1m-GFP-GUS*) was not (Figure S7). This suggests that the NBS-yB1a sequence is sufficient for NIN to activate transcription in *L. japonicus* roots.

The overlapping expression of both *NF-Y* genes in root nodule primordia suggests that LjNF-YA1 and LjNF-YB1 might function together in root nodule development. We examined whether LjNF-YA1 binds to LjNF-YB1. Since binding of NF-YA to NF-YB depends on dimerization of the latter protein with NF-YC [31], [44], we performed bimolecular fluorescence complementation (BiFC) analyses in *N. benthamiana* leaves. We expected that the LjNF-Y subunits might form heterotrimeric complexes with the endogenous tobacco NF-YC. eYFP signals were detected in nuclei when eYFP_C_-LjNF-YA1 (a fusion with the C-terminal half of eYFP) and eYFP_N_-LjNF-YB1 (a fusion with the N-terminal half of eYFP) were transiently co-expressed in tobacco leaves (Figure 3G). However, interactions were not observed between eYFP_C_-LjNF-YA1 and eYFP_N_-LjNF-YB2, containing the LjNF-YB1 homolog, LjNF-YB2 (see Figure S3B) (Figure 3H). On the other hand, eYFP_C_-LjNF-YA2 (containing the LjNF-YA1 homolog LjNF-YA2; see Figure S3A), interacted with eYFP_N_-LjNF-YB1 and with eYFP_N_-LjNF-YB2, but not with eYFP_N_ (Figure 3I–3K). eYFP signals were not detected when eYFP_N_-LjNF-YB1 and eYFP_C_ were co-expressed (Figure 3L). These results suggest that LjNF-YA1 binds to LjNF-YB1 in *planta*.

### Knockdown of *LjNF-YA1* prevents root nodule formation

**Figure 4:**
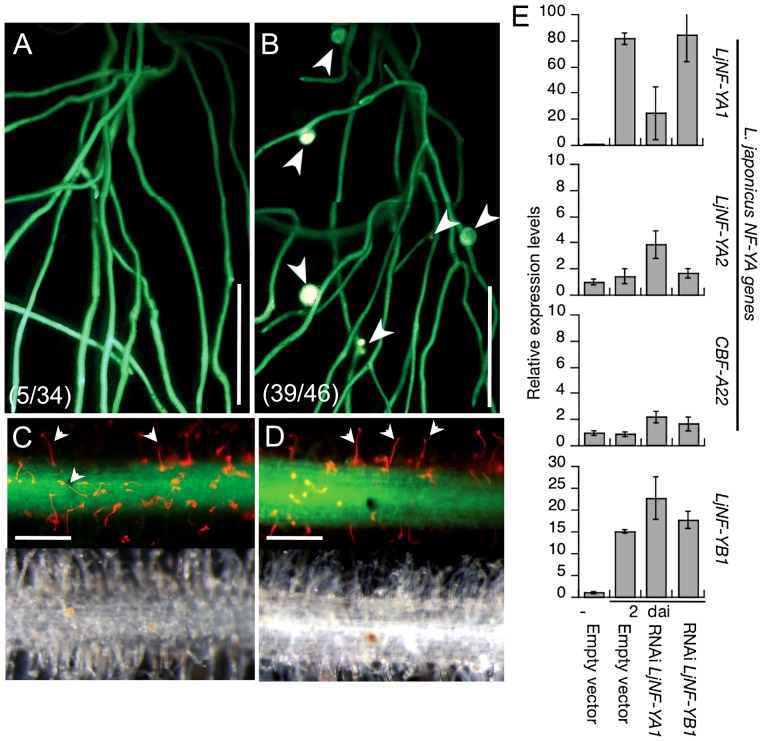
Inhibition of root nodule development by the knockdown of *LjNF-YA1*. Roots that were transformed with either *ProLjUb-RNAi-LjNF-YA1* (A,C) or an empty vector (B,D) were inoculated with DsRed-labeled *M. loti* for 14 days. (A,B) Root nodule formation in transformed roots. Fluorescence is visible from GFP (the root transformation marker) and from DsRed expressed in *M. loti*. Arrowheads indicate root nodules. The fractions of plants that formed root nodules on GFP-positive roots are shown in parentheses. (C,D) Infection thread formation in transformed roots. Fluorescence from GFP and DsRed is shown in the upper panels and bright field images are shown in the bottom panels of the same roots. Arrowheads indicate infection threads visualized by DsRed-labeled *M. loti*. Bars: 5 mm in (A,B), 0.2 mm in (C,D). (E) RT-PCR analyses of gene expression in roots that were transformed with either the empty vector, *ProLjUb-RNAi-LjNF-YA1*, or *ProLjUb-RNAi-LjNF-YB1*. Roots were inoculated with (2 dai; days after inoculation) or without (−) *M. loti*. Expression was analyzed for *LjNF-YB1* and three *L. japonicus* NF-Y subunit A genes (*LjNF-YA1*, *LjNF-YA2*, and *CBF-A22* [57]). The means and SDs from 3 biological repeats are shown.

To investigate loss-of-function phenotypes of *LjNF-YA1* and *LjNF-YB1*, RNAi constructs targeting the two genes were introduced into *L. japonicus* roots. These constructs were driven by the *LjUb* promoter. Although several *LjNF-YB1* RNAi constructs were tested, the gene was not downregulated (Figure 4E). On the other hand, an RNAi construct containing a sequence specific to the 3′-UTR of *LjNF-YA1* specifically prevented accumulation of *LjNF-YA1* mRNA (Figure 4E; Figure S3A). Therefore, a phenotypic analysis was performed using roots transformed with this *LjNF-YA1* RNAi construct. When inoculated with DsRed-labeled *M. loti* for 2 weeks, root nodules were produced in 85% of control plants that were transformed with the empty vector (Figure 4B). On the other hand, only 15% of plants with roots that were transformed with the *LjNF-YA1* RNAi construct generated infected root nodules (Figure 4A). The efficiency of root nodule formation was significantly reduced in roots transformed with the RNAi construct (Figure S8A). The numbers of small bumps without *M. loti* invasion were also decreased by the RNAi construct, suggesting that cortical cell division was inhibited at the early stages of the root nodule development. In contrast to root nodule organogenesis, infection threads were formed in 97% of the *LjNF-YA1* RNAi plants at efficiencies similar to those in control roots (Figure 4C, 4D; Figure S8A), indicating that epidermal responses resulting in infection thread growth were not influenced by the RNAi construct. Thus it appears that *LjNF-YA1* is required for the regulation of cortical cell division in the development of root nodules downstream of NIN. Consistent with this, the RNAi construct also prevented spontaneous nodule formation in gof-CCaMK roots [45] in the absence of *M. loti* (Figure S8B, S8C).

### *NIN* overexpression results in the formation of root nodule primordium-like structures

**Figure 5:**
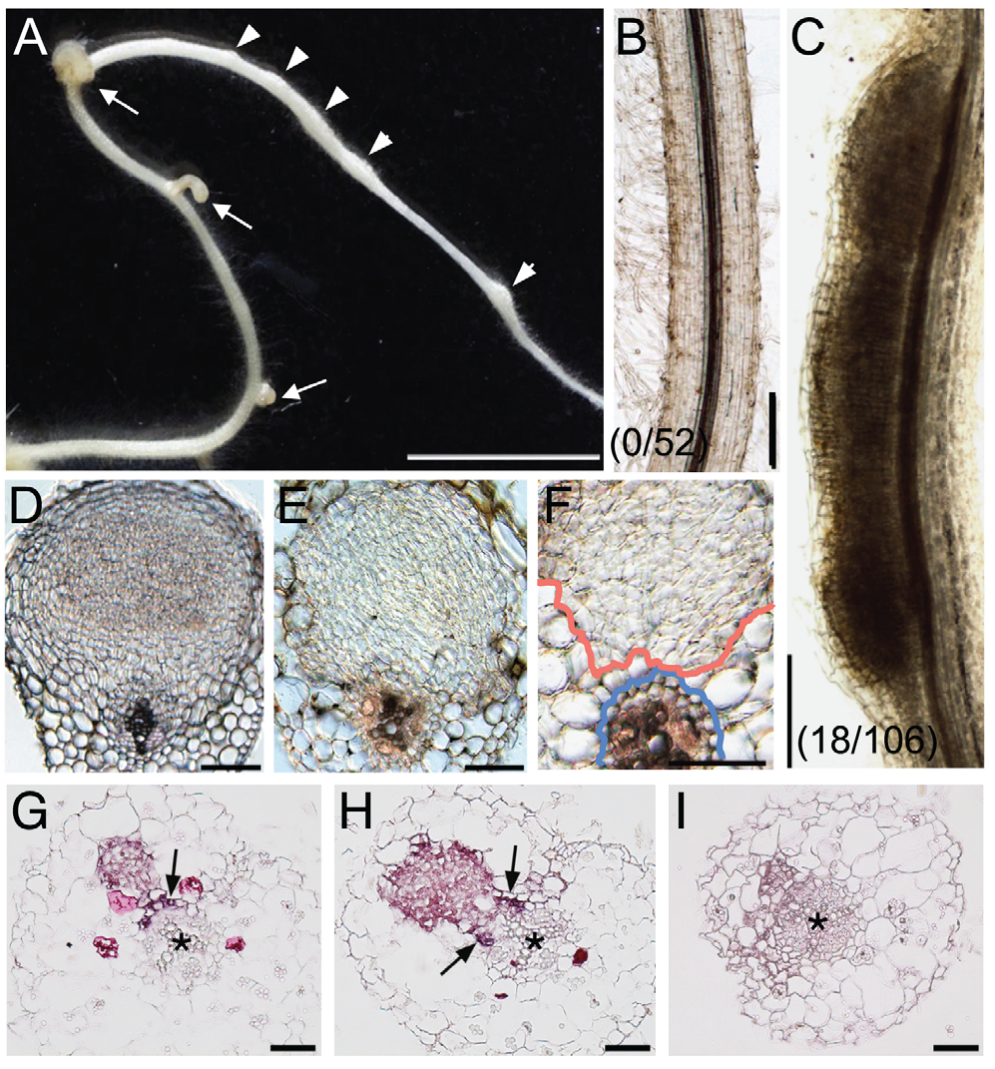
*NIN* overexpression induces cortical cell division. (A) A Gifu (wild-type) root was transformed with *ProLjUb-NIN* and then cultured for 6 weeks in the absence of *M. loti*. Bumps (arrowheads) and malformed lateral roots (arrows) are indicated. (B,C) Cleared roots that were transformed with either an empty vector (B) or *ProLjUb-NIN* (C). The fractions of plants with bumps are shown in parentheses. (D) A transverse section of a root nodule primordium (10 dai) formed on a MG-20 (wild-type) root that was transformed with the empty vector. (E,F) Transverse sections of bumps formed on uninoculated MG-20 roots that were transformed with *ProLjUb-NIN*. Blue and red lines in (F) represent the outer edges of the endodermis and the boundary of the region with dividing cortical cells, respectively. (G–I) *in situ* RNA hybridization of *ENOD40-1* in transverse sections of bumps caused by *NIN* overexpression, using either antisense (G,H) or sense probes (I). Asterisks indicate the central xylem. Arrows indicate the pericycle with *in situ* signals. Bars: 5 mm in (A); 0.2 mm in (B,C); 0.1 mm in (D–F); 50 µm in (G–I).

It has been shown that NIN is a nodulation-specific factor essential for root nodule organogenesis. The NIN-target gene *LjNF-YA1* is also required for root nodule organogenesis. To investigate whether NIN confers cortical cell division in the absence of *M. loti*, we produced *L. japonicus* roots that ectopically overexpressed *NIN* cDNA from the *LjUb* promoter (*ProLjUb-NIN*). In uninoculated *NIN*-overexpressing roots we found lateral roots with enlarged tips and bumps similar to root nodule primordia (Figure 5A, 5C). Such abnormalities were not observed in roots transformed with the empty vector (Figure 5B). In the *NIN*-overexpressing roots that exhibited the abnormal architecture, 74% (n = 7) of the lateral roots were malformed. The bumps occurred in roots with the abnormal lateral roots, and were often broader along the apical-basal axis than normal root nodules (Figure 5C; Figure S9). When a belt-shaped bump was counted as one, the mean bump number was 3.55±1.63 (n = 11) on roots that generated them. Microscopic analyses revealed that the bumps were formed via cortical cell division that preferentially occurred at positions opposite the protoxylem poles (6/8 bumps) (Figure 5E, 5F). This was also seen in wild-type root nodule formation (11/12 root nodules; Figure 5D). The NIN-induced bumps were anatomically similar to the root nodule primordia. Thus, NIN activity results in cortical cell division. However, unlike the infected wild-type root nodules the NIN-induced bumps did not develop peripheral vascular systems. NIN-induced bumps were also formed on transgenic roots carrying mutations in genes that function upstream of *NIN*, including *CCaMK*, *LHK1*, *NSP1*, and *NSP2* (Figure S10A–S10E). The lower efficiency of bump generation in *nsp2* mutants implies that NSP2 contributes to the regulation of cortical cell division independently of NIN.

We used *in situ* RNA hybridization to investigate expression of an early nodulin gene, *ENOD40-1*, in the NIN-induced bumps. This nodulin gene is often used as a molecular maker for rhizobial infection. High levels of *ENOD40-1* expression were detected in dividing cortical cells within the NIN-induced bumps, and in pericycle cells opposite to the xylem pole (Figure 5G, 5H). This expression pattern was similar to that seen in root nodules caused by Nod factors and rhizobial infection [46]–[48], indicating that the NIN-induced bumps possess a root nodule primordium-like identity at the molecular level.

### Overexpression of *NIN* and *NF-Y* subunit genes enhances cell division

**Figure 6:**
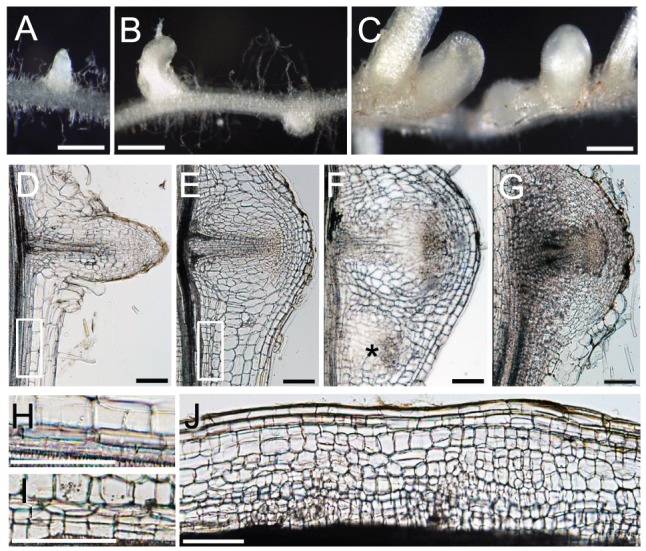
NIN and NF-Y subunits regulate cell division. (A–C) Lateral roots formed on MG-20 roots that were transformed with either an empty vector (A), *ProLjUb-LjNF-YA1* (B), or *ProLjUb-LjNF-YA1 Pro35S-LjNF-YB1* (C). Roots were cultured in the absence of *M. loti*. (D–G) Longitudinal sections of lateral roots formed on MG-20 roots that were transformed with either the empty vector (D), *ProLjUb-LjNF-YA1 Pro35S-LjNF-YB1* (E,F), or *ProLjUb-NIN* (G). An asterisk in (F) indicates an additional lateral root meristem-like structure. (H,I) Magnified images of the boxed regions in (D) and (E), respectively. (J) A longitudinal section of the root that was transformed with *ProLjUb-LjNF-YA1* and *Pro35S-LjNF-YB1*. Note the presence of small cells that were generated by cortical cell division. Bars: 1 mm in (A–C); 0.1 mm in (D–G,J); 0.2 mm in (H,I).

We next ectopically overexpressed the *LjNF-YA1* and *LjNF-YB1* cDNAs to examine whether the *Lotus* NF-Y subunits function to regulate cell proliferation. Roots overexpressing *LjNF-YA1* produced lateral roots with malformed tips (Figure 6B; Figure S11C, S11F), similar to those formed as a result of *NIN* overexpression (Figure 5A). Such abnormalities were not observed in roots transformed with either the empty vector or *ProLjUb-LjNF-YB1* alone (Figure 6A; Figure S11A, S11B, S11F). However, the co-expression of *LjNF-YB1* exaggerated the root architecture abnormalities caused by *LjNF-YA1* overexpression (Figure 6C; Figure S11D, S11F). This effect was not observed when *LjNF-YB2* was co-expressed with *LjNF-YA1* (Figure S11G–S11I), indicating a functional specificity of the interaction between LjNF-YA1 and LjNF-YB1.

The intervals between lateral roots were shorter in roots that co-overexpressed *LjNF-YA1* and *LjNF-YB1* (Figure 6C; Figure S11E). Extra cell division was observed in the lateral root primordia and their proximal regions, including the pericycle, which is the origin of the lateral root primordium (Figure 6D, 6E, 6H, 6I). This pattern was also observed in the *NIN*-overexpressing roots (Figure 6G). In the *LjNF-YA1* and *LjNF-YB1* co-overexpressing roots, additional lateral root meristem-like structures emerged in the proximal regions of the lateral root primordia (Figure 6F). Although abnormal cell division occurred in the cortex of these roots (Figure 6J), they did not form visible bumps similar to those found on roots overexpressing *NIN*. These results indicate that NIN and the NF-Y subunits positively influence cell division. The effect of *LjNF-YA1* overexpression is consistent with the role of *LjNF-YA1* in root nodule primordium development (Figure 4A).

To investigate cell division activities in non-meristematic regions of roots that were transformed with either *ProLjUb-NIN* or *ProLjUb-LjNF-YA1 Pro35S-LjNF-YB1*, we produced plant lines that were stably transformed with a cell division marker, *ProLjCycB1;1-CycB1;1(NT)-GUS*. In this construct, a 4 kb genomic fragment of *L. japonicus Cyclin B1;1* (chr1.CM0269.150.r2.m) was translationally fused to the *GUS* reporter coding sequence. This genomic fragment contains the 2.9 kb promoter region from the putative initiation codon and the coding region that corresponds to the N-terminal part of the protein including the destruction-box. This reporter showed dot-like expression patterns in meristem regions of root tips, lateral root primordia, and developing root nodules (Figure S12A–S12F). We introduced either *ProLjUb-NIN* or *ProLjUb-LjNF-YA1 Pro35S-LjNF-YB1* into roots of *ProLjCycB1;1-CycB1;1(NT)-GUS* plants. Ectopically localized GUS staining was detected in the cortex, pericycle, and endodermis of these roots, in addition to root tip regions and lateral root primordia (Figure S12G–S12I). The number of GUS staining foci was significantly increased as compared to control roots (Figure S12J). These results indicated that overexprression of *NIN* and the *NF-Y* subunit genes resulted in increase in division activity.

**Figure 7:**
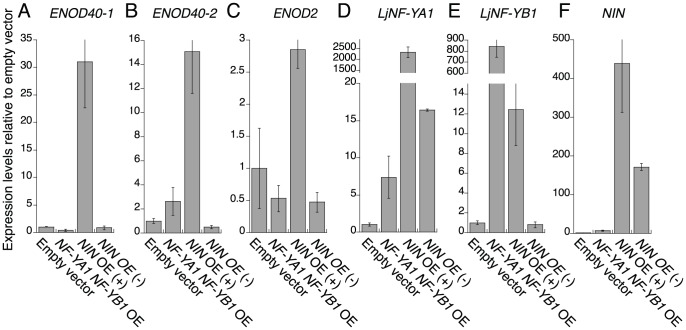
RT–PCR analysis of the expression of early nodulin genes and *LjNF-Y* subunit genes. Expression was analyzed for *ENOD40-1* (A), *ENOD40-2* (B), *ENOD2* (C), *LjNF-YA1* (D), *LjNF-YB1* (E), and *NIN* (F). Plants were cultured for 3 weeks without *M. loti* inoculation. Total RNA was isolated from roots transformed with either an empty vector, *ProLjUb-LjNF-YA1* plus *Pro35S-LjNF-YB1* (*NF-YA1 NF-YB1* OE), or *ProLjUb-NIN* (*NIN* OE). *ProLjUb-NIN* roots that exhibited altered structures (+) were harvested separately from roots with no morphological alterations (−). The means and SDs from 3 biological repeats are shown.

We analyzed expression of the early nodulin genes *ENOD40-1*, *ENOD40-2*, and *ENOD2* in roots overexpressing the *NF-Y* genes, and compared the results with those of roots transformed with *ProLjUb-NIN* or the empty vector. The expression of nodulin genes was upregulated in the *NIN*-overexpressing roots that had altered structures. However, nodulin gene expression levels were not significantly different from the vector control in either roots overexpressing both the *NF-Y* genes or roots overexpressing *NIN* but with no visible alterations in structure (Figure 7A, 7B, 7C, 7F). Thus, expression of the nodulin genes was correlated with alterations in root morphology in the *NIN*-overexpressing roots. The *NF-Y* genes did not upregulate expression of the nodulin genes, even if aberrant lateral roots were formed. This result indicates that the function of the *Lotus NF-Y* genes is not associated with root nodule primordium identity. We also found that *LjNF-YB1* expression was not upregulated in *NIN*-overexpressing roots with no visible alterations in root structure (Figure 7D, 7E). This result is consistent with the idea that expression of both the *NF-Y* genes is important to stimulate cell division.

## Discussion

We have characterized the molecular role of NIN as a transcriptional activator that directly targets the *NF-Y* subunit genes *LjNF-YA1* and *LjNF-YB1* (Figure 2). The DEX-inducible NIN protein induced expression of the *NF-Y* genes without *M. loti* inoculation (Figure 1). We further found that NIN has the ability to induce cortical cell division in the absence of bacterial symbionts (Figure 5). Functional analyses using both RNAi and overexpression indicated that the *NF-Y* genes play overlapping roles with that of *NIN* (Figure 4; Figure 6). It appears that the *Lotus* NF-Y subunits contribute to cortical cell division in a NIN-mediated transcriptional pathway.

### NIN is a key player in root nodule organogenesis

*NIN* overexpression induced bumps in the absence of *M. loti* that were anatomically similar to root nodule primordia (Figure 5). The overexpression of *M. truncatula response regulator9* induces arrested primordium-like structures in which cell division occurs in the pericycle, endodermis, and cortex [49]. Although these arrested primordia are somewhat similar to the bumps caused by *NIN* overexpression, the former structures are probably lateral root primordia [49]. In *L. japonicus* and *M. truncatula*, lateral root primordia originate from the pericycle and are also associated with cell division in the endodermis and cortex [49]. NIN-induced bumps, on the other hand, are initially generated by cortical cell division at a radial position where root nodule primordia are usually formed. Furthermore, *ENOD40* is expressed in NIN-induced bumps with patterns characteristics of root nodules (Figure 5). We conclude that the bumps caused by *NIN* overexpression possess a root nodule primordium-like identity. NIN is a factor that initiates cortical cell division during root nodule organogenesis. The NIN-induced bumps were formed on roots of the symbiotic mutants *ccamk-3*, *hit1*, *nsp1*, and *nsp2* (Figure S10), indicating that NIN functions downstream or independently of these symbiotic genes.

Bump formation resulting from *NIN* overexpression (17% of plants with transformed roots; Figure 5) is less efficient than the spontaneous root nodule formation observed in gof-CCaMK and gof-LHK roots (67 and 40%, respectively) [10]. Peripheral vascular systems were not formed in the NIN-induced bumps. Thus, it is unlikely that NIN is the sole factor regulating the initiation of cortical cell division and root nodule organogenesis. NSP2 is also required for root nodule organogenesis downstream of LHK1 [14]. We found that *NSP2* influenced the efficiency of bump formation in *NIN-*overexpressing roots (Figure S10). Cytokinin signaling is involved in developmental programs that are generally conserved in plant species. It is likely that LHK1 regulates such pathways in addition to those specific to nodulation. Alternatively, NIN itself may prevent the efficient initiation of cortical cell division. NIN acts as a negative regulator in some nodulation processes. For example, *M. truncatula NIN* negatively influences the spatial pattern of *ENOD11* expression [28]. Also in *M. truncatula*, a loss-of-function *NIN* mutation represses expression of the *CLE* genes, which encode small peptides that act in an inhibitory autoregulation pathway for root nodule formation [50]. Deregulated expression of *NIN* may activate this negative-feedback regulation system, reducing the efficiency of *NIN*-induced bump formation.

We also found that regions where the NIN-induced bumps emerged were often expanded along the apical-basal axis, whereas cortical cell division occurred at the correct radial position (Figure 5). These findings suggest that the radial position where cortical cell division occurs is defined independently of *NIN* expression. Regulatory mechanisms, by which *NIN* expression is restricted to the proper area along the apical-basal axis, are important for generating root nodule meristems of the correct size. The *M. truncatula* ethylene-insensitive *sickle* mutants form sequentially connected root nodules [51]. However, it is likely that the radial positioning of NIN-induced bumps is under the control of ethylene, because ethylene also influences radial positioning [52], [53]. *NIN* expression appears to be spatially regulated downstream of LHK1 activation. *NIN* expression was restricted to regions within or near dividing cortical cells in roots that were treated with exogenous cytokinin [18]. Feedback mechanisms may negatively influence the division of cortical cells surrounding root nodule primordia through modulation of the LHK1 signaling pathway.

### NF-Y subunits function downstream of NIN

We searched for genes whose expression was positively regulated by NIN using the transcriptome database, and found several candidates that might be targeted by NIN. We used ChIP assays and EMSAs to show that *LjNF-YA1* and *LjNF-YB1* are direct NIN targets (Figure 2), and then identified the NBSs in the promoters of these genes. Previously-identified regulatory elements that control expression of nodulation-related genes [30], [54]–[56] do not contains sequences similar to the NBSs. Therefore, the NBSs may be novel elements associated with nodulation.

The *Lotus NF-YA1* gene reported here is orthologous to *MtHAP2-1* (Figure S3A), and identical to *CBF-A01*, which is induced by *M. loti* inoculation [57]. The knockdown of *MtHAP2-1* resulted in an arrest of root nodule growth that was associated with the absence of a clear meristem zone [32]. This zone is usually present at the apical region of a tip-growing indeterminate-type root nodule. *MtHAP2-1* expression is restricted to this meristem zone by post-transcriptional regulatory mechanisms [32], [58]. The *Lotus* counterpart, on the other hand, is required for the regulation of cortical cell division during the early stages of determinate-type root nodule development (Figure 4; Figure S8). We showed that NIN transcriptionally regulates *LjNF-YA1* expression. The expression pattern is consistent with that of *NIN* and with the function of LjNF-YA1 in root nodule organogenesis. We further demonstrated that LjNF-YA1 interacts with LjNF-YB1. The overexpression of *LjNF-YB1* exaggerated the effect of *LjNF-YA1* overexpression on cell division (Figure 6; Figure S11). Our results, showing expression of both genes in the root nodule primordia and interactions between the proteins *in planta* (Figure 3), support the idea that the *Lotus* NF-Y subunits function in the same NF-Y complex during root nodule development. Thus, the two NF-Y proteins participate in the stimulation and/or promotion of cell division.

While *LjNF-YA1* overexpression influenced the root architecture, *LjNF-YB1* overexpression alone did not affect cell division. Similarly, overexpression of the common bean *NF-YC1* did not influence root architecture apart from an increase in root nodule number [38]. Therefore, *LjNF-YA1* expression is of primary importance for the control of cell division. A phylogenetic analysis suggests that the *NF-YA* genes in the clade that includes *LjNF-YA1* and *MtHAP2-1* have evolved as a result of duplication in the ancestral legume lineage (Figure S3). Interestingly, the expression of *NF-YA* genes that are related to *LjNF-YA1* and *MtHAP2-1* is strongly induced in actinorhizal root nodules, which are lateral root-like structures generated by interactions between the non-legume plants *Casuarina glauca* and *Alnus glutinosa* and their *Frankia* symbionts [59]. This implies that NF-Y is important for root nodule organogenesis in actinorhizal plants, and leads to the speculation that different types of root nodule symbiosis system recruited the molecular networks regulating the expression of *NF-Y* genes.

NF-Y complexes are general transcription factors that target CCAAT boxes. They regulate gene expression by influencing histone modifications [60]–[62]. Transcriptional activators, which work together with the NF-Y complexes, are required for the efficient expression of target genes [60], [63], [64]. The genes encoding such factors may be included among the NIN-target genes. Overexpression of the *Lotus NF-Y* subunit genes stimulated cell division in the lateral root primordia, resulting in the production of malformed lateral root tips at high frequencies. The ectopically expressed NF-Y subunits apparently interacted with factors other than NIN to stimulate the proliferation of cells with the competence for division in the lateral root primordia. Cortical cells, on the other hand, possess low meristematic activity in the absence of NIN activity. Factors that may act downstream of NIN would be required for the *Lotus* NF-Y subunits to fully stimulate cortical cell division.

### NIN functions in the establishment of the root nodule symbiosis system

Rice, which engages in mycorrhizal but not root nodule symbiosis, has genes corresponding to *NSP1*, *NSP2*, and the common *SYM* genes [65], [66]. These are functionally equivalent to the corresponding genes in *L. japonicus*, whereas the closest *NIN* homolog in rice, *OsNLP1*, does not rescue the *nin-2* phenotype [66]. NIN and its orthologous proteins in other legume species possess common structural characteristics distinct from other NIN-like proteins [33], [67], suggesting that NIN has acquired functions specialized for nodulation. NF-Y complexes, on the other hand, are transcription factors that are widespread among eukaryotes. The induction of extra cell division by the overexpression of the NF-Y subunits in lateral root primordia was likely due to the formation of complexes with other subunits that are expressed in dividing cells and/or cells with the competency to divide. This implies that NF-Y may be generally important for regulating cell division in plants, as it is in mammals. Importantly, overexpression of the common bean *NF-YC1* upregulated the expression of genes that encode cell cycle regulators [38]. The *NF-YC1* gene is ubiquitously expressed in various plant organs. NIN is thought to coordinate gene expression for onset of root nodule organogenesis by regulating the expression of genes encoding NF-Y components in addition to genes that are specifically involved in the regulation of cortical cell division. Unlike the results from overexpression of *NIN* and the *Lotus NF-Y* subunit genes, roots expressing gof-CCaMK have not been reported to show abnormally stimulated cell division during lateral root development [10]. This suggests that NIN and the NF-Y subunits are more directly involved in the regulation of cell division than CCaMK. NIN function may be related to the regulation of gene expression networks that are generally required for plant life, particularly those involved in cell division. Arabidopsis RKD proteins also stimulate cell division and possess the RWP-RK domain, suggesting that proteins containing this domain may have a general role in the transcriptional regulation of genes responsible for cell division [68], [69].

The evolution of NIN, which is thought to be specific to legume plants, may have led to the coordinated expression of the subset of genes that are involved in the generation of functionally *de novo* organs, the root nodules. A number of possible origins, including lateral roots, have been proposed for root nodules [70]. Our results are suggestive of common regulatory mechanisms that regulate cell proliferation during root nodule and lateral root organogenesis. A comprehensive analysis of the gene expression networks that are regulated by NIN would provide further clues about the root nodule-specific pathways that lead to the establishment of the root nodule symbiosis system.

## Materials and Methods

### Plant materials and bacterial strains

We used the *L. japonicus* accessions Gifu B-129 and MG-20 as wild-type plants, and the symbiotic mutants *nin-2* [19], *nsp1* (SL1795-4) [22], *nsp2* (*sym70*) [23], *ccamk-3* [13], *hit1* [15], and *cyclops*-3 [45]. These plants were inoculated with *M. loti* strains MAFF303099 or one that constitutively expresses DsRed [29]. Unless otherwise indicated in a figure legend, Gifu B-129 was used as the wild type. Root transformations with *A. rhizogenes* and inoculation procedures with *M. loti* were performed as described previously [71]. An empty vector with the GFP marker for selection was used as the controls in root transformations.

### Plasmid construction

Plasmids were constructed using standard molecular biology techniques. The *LjNF-YA1* and *LjNF-YB1* promoters were amplified by PCR from Gifu B-129 genomic DNA. The amplified fragments were inserted into an entry vector, pENTR1A (Invitrogen), that was digested with BamHI and NotI. The promoters were then transferred into pMDC162-GFP, which was produced by replacing the hygromycin resistance gene of pMDC162 [72] with *GFP*. PCR-amplified cDNAs of *NIN*, *LjNF-YA1*, *LjNF-YB1*, *LjNF-YA2*, and *LjNF-YB2* were digested using restriction enzyme sites in the linker sequences, and the resulting fragments were cloned into pENTR-1A. To produce the GFP, GAL4DBD, GR, and myc fusions of NIN, each cDNA was inserted into the XhoI site of pENTR1A downstream of the *NIN* cDNA lacking a stop codon (inserted between the KpnI and NotI sites). For the BiFC analysis, cDNAs for eYFP_N173_-myc (tagged with the myc epitope from pSPYNE(R)173 [73]) and eYFP_C155_-HA (tagged with the HA epitope from pSPYCE(MR) [73]) were amplified by PCR. The former fragment was inserted upstream of the *LjNF-YB1* and *LjNF-YB2* cDNAs in pENTR1A. The latter fragment was inserted upstream of the *LjNF-YA1* and *LjNF-YA2* cDNAs. For controls, *eYFP_N173_-myc* and *eYFP_C155_-HA* were cloned into pENTR1A. The *NIN*, *NIN-myc*, *LjNF-YA1*, and *LjNF-YB1* cDNAs were transferred from the ENTRY vectors into pUB-GW-GFP [43]; the *NIN*, *NIN-GFP*, *NIN-GAL4DBD*, *NIN-myc*, *GAL4DBD*, *eYFP_N173_-LjNF-YB1*, *eYFP_N173_-LjNF-YB2*, *eYFP_ N173_-myc*, *eYFP_ C155_-LjNF-YA1*, *eYFP_ C155_-LjNF-YA2*, and *eYFP_C155_-HA* cDNAs were transferred into pUB-GW-Hyg [43]; and the *NIN-GFP, NIN-GR*, and *NIN-myc* cDNAs were transferred into ProNIN-DC-NINter [66]. To co-express *LjNF-YA1* and *LjNF-YB1* in *L. japonicus* roots, the *LjNF-YA1* cDNA was transferred into pUB-GW-LjNF-YB1, which was produced by replacing the *GFP* gene in pUB-GW-GFP with the *LjNF-YB1* cDNA. For the knockdown analyses of *LjNF-YA1* and *LjNF-YB1*, the 3′-UTR of each gene was amplified by PCR, and cloned into pENTR/D-TOPO with reverse direction. The fragments were transferred into pUB-GWS-GFP [43]. For transcriptional activation analyses, 4xUAS with the *CaMV35S* minimal promoter was amplified by PCR from pTA7001 [74], and cloned into pENTR1A. 4xyB1a and 4xyB1m were synthesized by PCR and inserted between the SalI and BstXI sites of pENTR1A, with the *CaMV35S* minimal promoter between the NotI and XhoI sites. These synthetic promoters were transferred into pKGWFS7 [75]. For *in vitro* translation, cDNAs corresponding to *NIN-myc* and *NIN(520–878)-myc* were digested with SgfI and PmeI, and cloned into the pF3K-WG (BYDV) Flexi Vector (Promega). For *ProLjCycB1;1-CycB1;1(NT)-GUS*, the approximately 4 kb *LjCycB1;1* genomic fragment was amplified from Gifu B-129 genomic DNA. The genomic fragment was subcloned into pCR-Blunt (Invitrogen), and the fragment between the HindIII and SmaI sites was then inserted upstream of the *GUS* gene in the binary vector pBI101.3. The sequences of primers used for plasmid construction are shown in Table S1.

### GUS staining assays

Roots were washed with 100 mM NaPO_4_ buffer (pH 7.0), and incubated in GUS staining solution [100 mM NaPO_4_ (pH 7.0), 10 mM EDTA, 0.5 mg/ml 5-bromo-4-chloro-3-indolyl-b-glucuronic acid, 2 mM K_4_Fe(CN)_6_, 2 mM K_3_Fe(CN)_6_, and 0.1% Triton X-100] for 2 to 4 h at 37°C after vacuum infiltration for 10 min. To examine the transcriptional activity of NIN with the synthetic *4xyB1a* and *4xyB1m* promoter constructs, discs were cut out from *N. benthamiana* leaves 3 days after *Agrobacterium* infiltration, incubated in the GUS staining solution for 8 h, and then decolorized with 75% ethanol.

### Microscopic analysis

Observations of roots inoculated with DsRed-labeled *M. loti* were performed as described previously [10]. For the BiFC and transcriptional activation analyses, *N. benthamiana* leaves were observed 3 days after *Agrobacterium* infiltration. Confocal microscopy was performed using a μRadiance confocal microscope (BioRad). GFP and YFP florescence was induced using a 488 nm argon laser and imaged using an HQ530/60 filter. Autofluorescence from chloroplasts was induced using a 514 nm green HeNe laser and imaged using an E600LP emission filter.

### Expression analyses

*In situ* hybridizations with digoxigenin-labeled RNA probes was performed as described previously [47]. For RT-PCR, total RNAs were isolated from *L. japonicus* roots using the Plant RNeasy Mini kit (Qiagen). First strand cDNAs were synthesized using the QuantiTect Reverse Transcription kit (Qiagen). RT-PCR was performed in a LightCycler with the LightCycler FastStart DNA Master SYBR Green I reaction mix (Roche Applied Science). Expression levels were normalized using *polyubiquitin* transcripts. Primers for *polyubiquitin*, *NIN*, *LjENOD40-1*, *LjENOD40-2*, and *LjENOD2* were synthesized as described previously [19], [48], [76]. Sequences of the other primers used for RT-PCR are shown in Table S1.

### Electrophoresis mobility shift assays (EMSAs)

The NIN-myc and NIN(520–878)-myc proteins were synthesized using the TNT SP6 High-Yield Wheat Germ Protein Expression System (Promega). The *in vitro* translation products produced without templates were used as the control. Purified probes (10–30 fmol) were end-labeled with γ^32^P-ATP and incubated with the *in vitro* translation products (1.5–3 µl) in 20 µl of EMSA DNA binding buffer [20 mM Hepes-KOH (pH 7.9), 50 mM KCl, 1 mM MgCl_2_, 1 mM DTT, 4% glycerol, 0.1% Triton X-100] supplemented with 2 µg BSA and 400 ng dIdC, for 30 min at room temperature. Reactants were separated in 5% polyacrylamide gels with 0.5× TBE buffer at 150 V for 90 min. Radioactivity was visualized with an imaging analyzer (BAS2500; Fujifilm). The oligonucleotides that were used as probes and as primers for probe synthesis are shown in Table S1.

### ChIP assays

ChIP was carried out as described previously [77] with minor modifications. Roots were transformed with either *ProLjUb-NIN-myc* or the empty vector then fixed with 1% formaldehyde in MC buffer for 10 min under a vacuum. The reaction was stopped by adding 0.125 M glycine, and the roots were washed three times with MC buffer. The fixed tissue (0.7–1.0 g) was powdered with a mortar and pestle in liquid nitrogen, suspended with 15 ml of M1 buffer supplemented with 1 mM PMSF and Complete Protease Inhibitor Cocktail (Roche Diagnostics), and incubated for 30 min on ice. The crude extract was filtered through two layers of Miracloth and washed with 15 ml of M1 buffer. The filtrate was centrifuged at 1,600×g for 15 min at 4°C. The pellet was washed 4 times, each with 1 ml of M2 buffer, and once with M3 buffer. After centrifugation at 2,000×g for 10 min, the pellet was resuspended in 1 ml of Sonication buffer that was supplemented with 1 mM PMSF and Protease Inhibitor Cocktail, incubated for 20 min on ice, and sonicated 9 times for 10 seconds using a Branson Sonifier 250 at 40% duty cycle, power setting 4. The chromatin suspension was centrifuged 2 times at 14,000×g for 15 min at 4°C. An equal volume of IP buffer was added to the supernatant, and 50 µl were removed to use as the as Input. Then 2 µg of anti-myc polyclonal antibodies (Santa Cruz Biotechnology, Inc) were added to the remaining suspension and the mixture was incubated for 3 h at 4°C. After centrifugation, 20 µl of proteinA-agarose (25% slurry; Santa Cruz Biotechnology, Inc) that had been pre-incubated with 0.5 mg/ml sheared salmon sperm DNA was added to the supernatant, and this mixture was rotated for 1 h at 4°C. After washing 4 times with IP buffer, the immunoprecipitate was eluted twice with 100 µl of elution buffer, then 150 µl of 1 M Tris buffer (pH 9.0) was added into the combined eluates. The DNA reverse cross-linking procedure was performed as described previously [77]. After ethanol precipitation, the DNA was dissolved in 50 µl of 5 mM Tris-HCl (pH 8.0). RT-PCR was performed to determine whether the target promoter regions had been enriched. The PCR products were quantified by comparison with products amplified using primers specific to the 5S rRNA gene. The sequences of primers used to amplify promoter fragments from *LjNF-YA1*, *LjNF-YB1*, the rRNA gene, chr4.CM0179.190.r2.m, and chr6.CM1757.140.r2.m are shown in Table S1.

### Accession numbers

The sequences of genes listed in Figure S2A can be found at http://www.kazusa.or.jp/lotus/.

## Supporting Information

Figure S1 NIN acts as a transcriptional activator. (A,B) Confocal images of tobacco leaf epidermal cells that were transformed with either *ProLjUb-NIN-GFP* (A) or *Pro35S-GFP* (B). Fluorescent images of GFP are shown in the left panels and the same images overlaid with bright field images are shown in the right panels. (C) Suppression of the infection thread-defective *nin-2* phenotype by *ProNIN-NIN-GFP*. A bright field image of a transformed root is shown on the right and the fluorescent image of the DsRed in *M. loti* is shown on the left. Arrowheads indicate infection threads showing DsRed fluorescence. NIN-GFP conferred infection threads on *nin-2* mutants (14 dai), although GFP signals were not detected. (D,F) Confocal images of tobacco leaves, into which a *4xUAS-GFP-GUS* reporter was co-introduced with effectors *ProLjUb-NIN-GAL4DBD* (D), *ProLjUb-GAL4DBD* (E), or *ProLjUb-NIN-myc* (F). GFP fluorescence (green) and autofluorescence from chloroplasts (red) are shown. Bars: 20 µm in (A,B), 0.2 mm in (C), 0.1 mm in (D–F).
(TIFF)

Figure S2 Genes whose expression is upregulated by *M. loti* infection depending on *NIN*. (A) A list of NIN-target candidates. The *Lotus* transcript profiling resource (http://cgi-www.cs.au.dk/cgi-compbio/Niels/index.cgi) was used to search for the NIN-target candidates. Thirty identifiers from 44,040 probe sets were extracted with the following two criteria: 1) Expression levels upregulated over 3-fold in wild-type plants by both inoculation with *M. loti* and Nod factor treatment. 2) The ratio of induction by *M. loti* inoculation in wild-type plants was over 3-fold higher than in the *nin* mutant. Then, 19 genes that showed expression profiles similar to that of *NIN* in different conditions were selected. DNA sequences of the genes listed in the table can be found at http://www.kazusa.or.jp/lotus/. Annotations of the following proteins were cited from elsewhere: CBF-A01 [57], LjLYS3 [78]. (B) RT-PCR analysis of NIN-dependent expression of candidate genes. Total RNAs were prepared from roots of Gifu (wild-type; red) and *nin-2* (blue) that were inoculated either with or without *M. loti* for one day. The means and SDs from 3 biological repeats are shown.
(TIFF)

Figure S3 Phylogenetic trees of NF-Y subunit A and B proteins. The evolutionary histories of NF-YA (A) and NF-YB (B) proteins from non-legumes [*Arabidopsis thaliana* (At), *Cucumis sativus* (Cs), *Fragaria vesca* (Fv)], and legumes [*Glycine max* (Gm), *L. japonicus* (Lj), *M. truncatula* (Mt), and *Phaseolus vulgaris* (Pv)] were inferred using the Minimum Evolution method [79]. The trees are drawn to scale, with branch lengths in the same units as those of the evolutionary distances used to infer the phylogenetic tree. Evolutionary analyses were conducted in MEGA5 [80]. Clades containing LjNF-YA1 and LjNF-YB1 are indicated by blue boxes. NF-Y proteins of *G. max*, *M. truncatula*, and *P. vulgaris* in these clades are shown. Red underlines indicate *Lotus* NF-Y proteins we analyzed.
(TIFF)

Figure S4 Suppression of the infection thread-defective phenotype of *nin-2* mutants. *nin-2* roots were transformed with either *ProNIN-NIN-GR* (A,B) or *ProNIN-NIN-myc* (C), and inoculated with DsRed-labeled *M. loti* for 2 weeks. *ProNIN-NIN-GR* rescued the infection thread-defective phenotype depending on DEX. Arrowheads and arrows indicate microcolonies and infection threads visualized by DsRed. These *NIN* constructs suppressed the defect in infection thread development. However, the defect in root nodule organogenesis was not rescued. Bar: 20 µm.
(TIFF)

Figure S5 NIN binds to specific nucleotide sequences in promoter regions of NIN-target genes. (A–D) Identification of NIN-binding nucleotide sequences in the *LjNF-YB1* promoter. (A) Probes used for EMSA in (B–D). (a) Green and red lines represent probes for the *LjNF-YB1* promoter shown in Figure 2A (probe 9 and 10) and those used in (B), respectively. (b) A nucleotide sequence of probe 14 and nucleotide substitutions in probes m1–14 are shown. The underline indicates probe NBS-yB1a. (B–D) EMSA using NIN(520–878)-myc. Arrowheads indicate mobility-shifted probes due to binding of the NIN protein. (B) Probes shown in (Aa) were examined. (C) Probe 14 and its derivatives (m1–5) were examined. (D) Probe NBS-yB1a and its derivatives (m6–14) were examined. Note that nucleotide substitutions in m3, m5, m8, and m12–14 abolished NIN-binding, while those in m4, m7, and m11 diminished the probes' affinities to NIN. (E) Competition assay using the NIN-target nucleotide sequences that were found in the *LjNF-YB1* and *LjNF-YA1* promoters. The NIN protein and labeled probe NBS-yB1a were incubated with different amounts of unlabeled competitors, NBS-yB1a, -yB1b, and -yA1, and m12. Relative band intensities are shown at the bottom of lanes.
(TIFF)

Figure S6 NIN binds to the promoter of chr4.CM0179.190.r2.m *in vivo*. ChIP analysis of chromatin suspensions that were prepared from roots transformed with either *ProLjUb-NIN-my*c or an empty vector. chr6.CM1757.140.r2.m is a control for RT-PCR. The levels of enrichment compared with those obtained from the empty vector are shown. Data are means and SDs from 3 biological repeats.
(TIFF)

Figure S7 Expression of GUS in developing root nodules. Roots were transformed with either *4xyB1a-GUS* (A) or *4xyB1m-GUS* (B) and inoculated with *M. loti*. Bars: 0.2 mm
(TIFF)

Figure S8 Phenotype of roots transformed with the *LjNF-YA1* RNAi construct. (A) Quantitative analyses of infection threads (ITs; black bars), bumps (white), and root nodules (gray) formed on roots that were transformed with either an empty vector (n = 13) or *ProLjUb-RNAi-LjNF-YA1* (n = 13). Plants were inoculated with *M. loti* for 14 days. (B,C) Suppression of spontaneous root nodule formation by *LjNF-YA1* RNAi. Roots transformed with either the empty vector (B) or *ProLjUb-RNAi-LjNF-YA1* (C) were generated from stably transformed plants carrying *Pro35S-CCaMK^T265D^*, and cultured in the absence of *M. loti*. Fluorescent images of GFP as a transformation marker are shown in the left panels and bright field images are shown on the right. Arrowheads indicate spontaneous root nodules formed on roots without GFP expression. The fractions of plants that formed spontaneous root nodules in roots with GFP expression are shown in parentheses. Bars: 2 mm.
(TIFF)

Figure S9 Bumps in a *nin-2* root that was transformed with *ProLjUb-NIN*. The plant was cultured for 7 weeks after transformation in the absence of *M. loti*. Bar: 2 mm.
(TIFF)

Figure S10 Bump formation in symbiotic mutants caused by *NIN* overexpression. (A–E) Bumps formed on roots of *ccamk-3* (A), *hit1* (B), *nsp2* (C), and *nsp1* (D) are shown. Roots were cultured in the absence of *M. loti* for 6 weeks. (E) Quantitative analysis of bump formation caused by *NIN* overexpression. The “Plant number” column shows the fractions of plants that formed bumps. The *P*-values from Fisher's exact test comparing data with those from the Gifu plants are shown in parentheses. The mean numbers of bumps per root and SDs are shown in the third column, with numbers of analyzed roots in parentheses. Bars: 0.1 mm in (A–D).
(TIFF)

Figure S11 Phenotypes of roots overexpressing *NF-Y* subunit genes. (A–D) Roots that were transformed with either an empty vector (A), *ProLjUb-LjNF-YB1* (B), *ProLjUb-LjNF-YA1* (C), or *ProLjUb-LjNF-YA1 Pro35S-LjNF-YB1* (D). The fractions of plants with malformed lateral roots are shown in parentheses. For (A–C), plants showing expression of a GFP selection marker were analyzed. For (D), plants that generated hairy roots were analyzed, because the selection marker was substituted by the *LjNF-YB1* cDNA in the double overexpression construct. (E) Quantifications of lateral roots formed on roots that were transformed with the empty vector (n = 29), *ProLjUb-LjNF-YA1* (n = 33), *ProLjUb-LjNF-YB1* (n = 33), or *ProLjUb-LjNF-YA1 Pro35S-LjNF-YB1* (n = 35). The means and SDs are shown. (F) Proportions of lateral roots with malformed tips in roots transformed with the empty vector (n = 8), *ProLjUb-LjNF-YA1* (n = 8), *ProLjUb-LjNF-YB1* (n = 6), or *ProLjUb-LjNF-YA1 Pro35S-LjNF-YB1* (n = 16). The means and SDs are shown. (G–I) Roots transformed with either *ProLjUb-LjNF-YA1 Pro35S-LjNF-YB2* (G,I) or the empty vector (H). Bars: 5 mm in (A–D,G); 1 mm in (H,I).
(TIFF)

Figure S12 Expression of *ProLjCycB1;1-CycB1;1(NT)-GUS* in *L. japonicus* roots. (A–D) GUS staining in a primary root tip (A,B), a lateral root primordium (C), and a root nodule primordium (D) are shown. (B) Higher magnification of (A). Asterisks indicate GUS staining in adjacent daughter cells. Note that *ProLjCycB1;1-CycB1;1(NT)-GUS* shows typical dot-like expression patterns of *Cyclin B1* as observed in Arabidopsis and soybean [81], [82]. (E,F) GUS staining in cortical cells beneath a root hair cell that was infected by *M. loti*. Images in (E) and (F) are the same root region focused on the epidermis (E) and the cortex (F). Arrows indicate a position of a curled root hair. (G–I) *ProLjCycB1;1-CycB1;1(NT)-GUS* expression in roots that were transformed with either an empty vector (G), *ProLjUb-NIN* (H), or *ProLjUb-LjNF-YA1 Pro35S-LjNF-YB1* (I). Roots were cultured on agar media for 16 days in the absence of *M. loti*. (J) The number of GUS foci in cortical layers of roots that were transformed with indicated constructs. Bars: 50 µm in (A–F), 100 µm in (G–I).
(TIFF)

Table S1 Sequences of oligo-nucleotides used for primers and EMSA probes. Primer and probe sequences used in the experiments are shown. Construction of plasmids (A), Gene expression analysis (B), ChIP analysis and EMSA (C).
(PDF)
