# Supplementary figures and images for: NODULE INCEPTION Directly Targets NF-Y Subunit Genes to Regulate Essential Processes of Root Nodule Development in Lotus japonicus
Source: PLoS Genet. 2013 Mar 21;9(3):e1003352. doi: 10.1371/journal.pgen.1003352 (PMC3605141; doi:10.1371/journal.pgen.1003352)

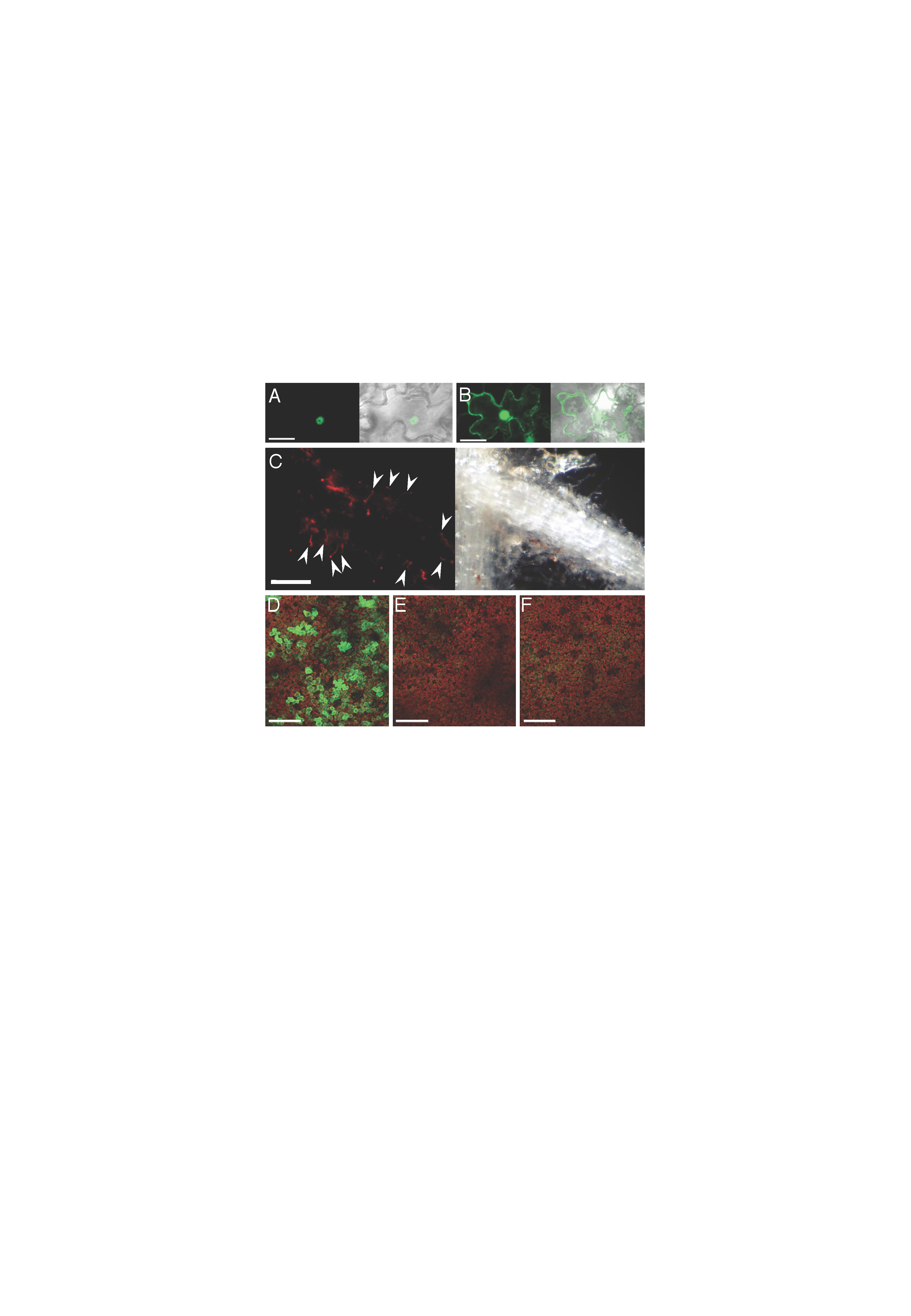

Supplement: Figure S1 — NIN acts as a transcriptional activator. (A,B) Confocal images of tobacco leaf epidermal cells that were transformed with either ProLjUb-NIN-GFP (A) or Pro35S-GFP (B). Fluorescent images of GFP are shown in the left panels and the same images overlaid with bright field images are shown in the right panels. (C) Suppression of the infection thread-defective nin-2 phenotype by ProNIN-NIN-GFP. A bright field image of a transformed root is shown on the right and the fluorescent image of the DsRed in M. loti is shown on the left. Arrowheads indicate infection threads showing DsRed fluorescence. NIN-GFP conferred infection threads on nin-2 mutants (14 dai), although GFP signals were not detected. (D,F) Confocal images of tobacco leaves, into which a 4xUAS-GFP-GUS reporter was co-introduced with effectors ProLjUb-NIN-GAL4DBD (D), ProLjUb-GAL4DBD (E), or ProLjUb-NIN-myc (F). GFP fluorescence (green) and autofluorescence from chloroplasts (red) are shown. Bars: 20 µm in (A,B), 0.2 mm in (C), 0.1 mm in (D–F). (TIFF) [file pgen.1003352.s001.tif]

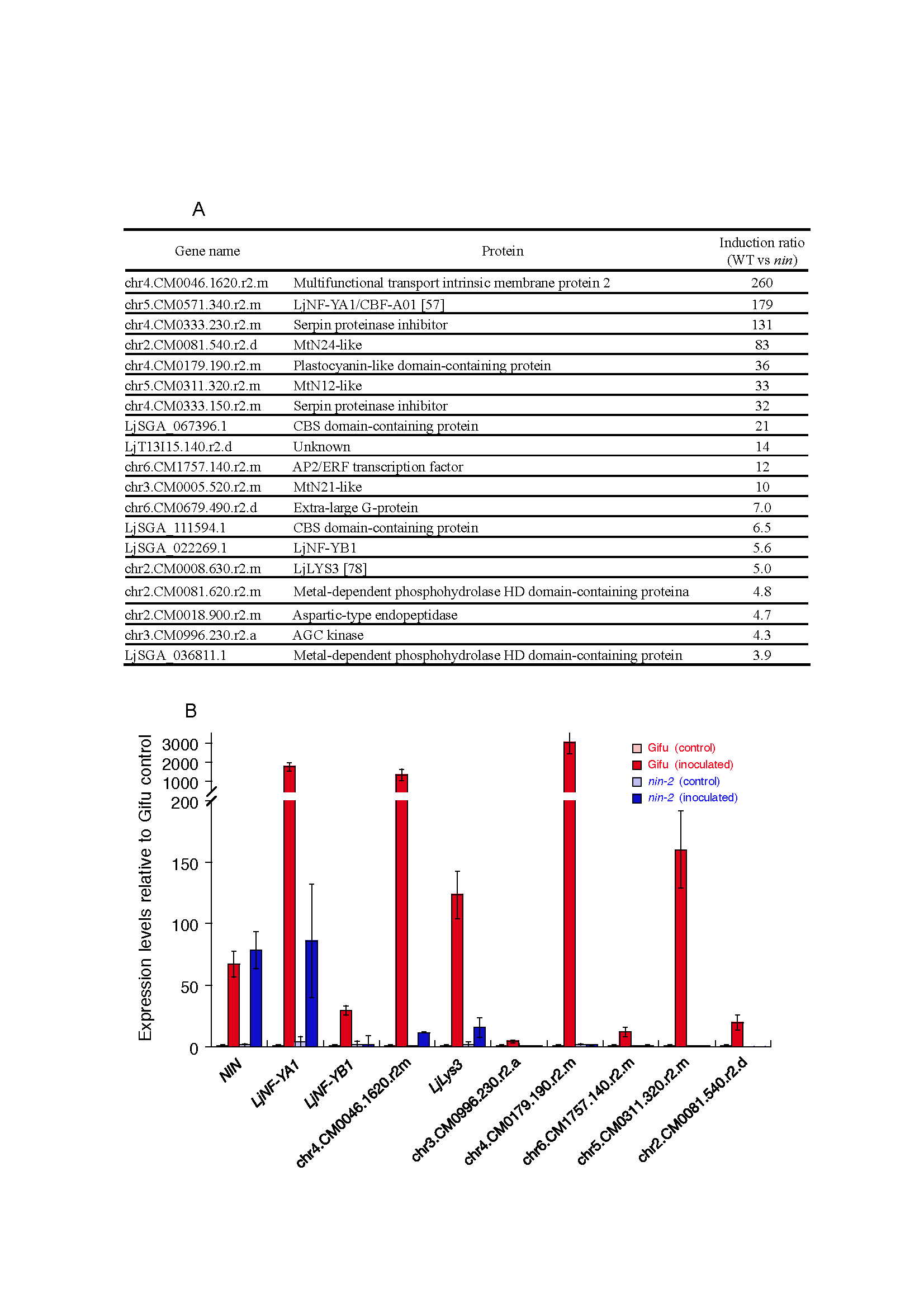

Supplement: Figure S2 — Genes whose expression is upregulated by M. loti infection depending on NIN. (A) A list of NIN-target candidates. The Lotus transcript profiling resource (http://cgi-www.cs.au.dk/cgi-compbio/Niels/index.cgi) was used to search for the NIN-target candidates. Thirty identifiers from 44,040 probe sets were extracted with the following two criteria: 1) Expression levels upregulated over 3-fold in wild-type plants by both inoculation with M. loti and Nod factor treatment. 2) The ratio of induction by M. loti inoculation in wild-type plants was over 3-fold higher than in the nin mutant. Then, 19 genes that showed expression profiles similar to that of NIN in different conditions were selected. DNA sequences of the genes listed in the table can be found at http://www.kazusa.or.jp/lotus/. Annotations of the following proteins were cited from elsewhere: CBF-A01 [57], LjLYS3 [78]. (B) RT-PCR analysis of NIN-dependent expression of candidate genes. Total RNAs were prepared from roots of Gifu (wild-type; red) and nin-2 (blue) that were inoculated either with or without M. loti for one day. The means and SDs from 3 biological repeats are shown. (TIFF) [file pgen.1003352.s002.tif]

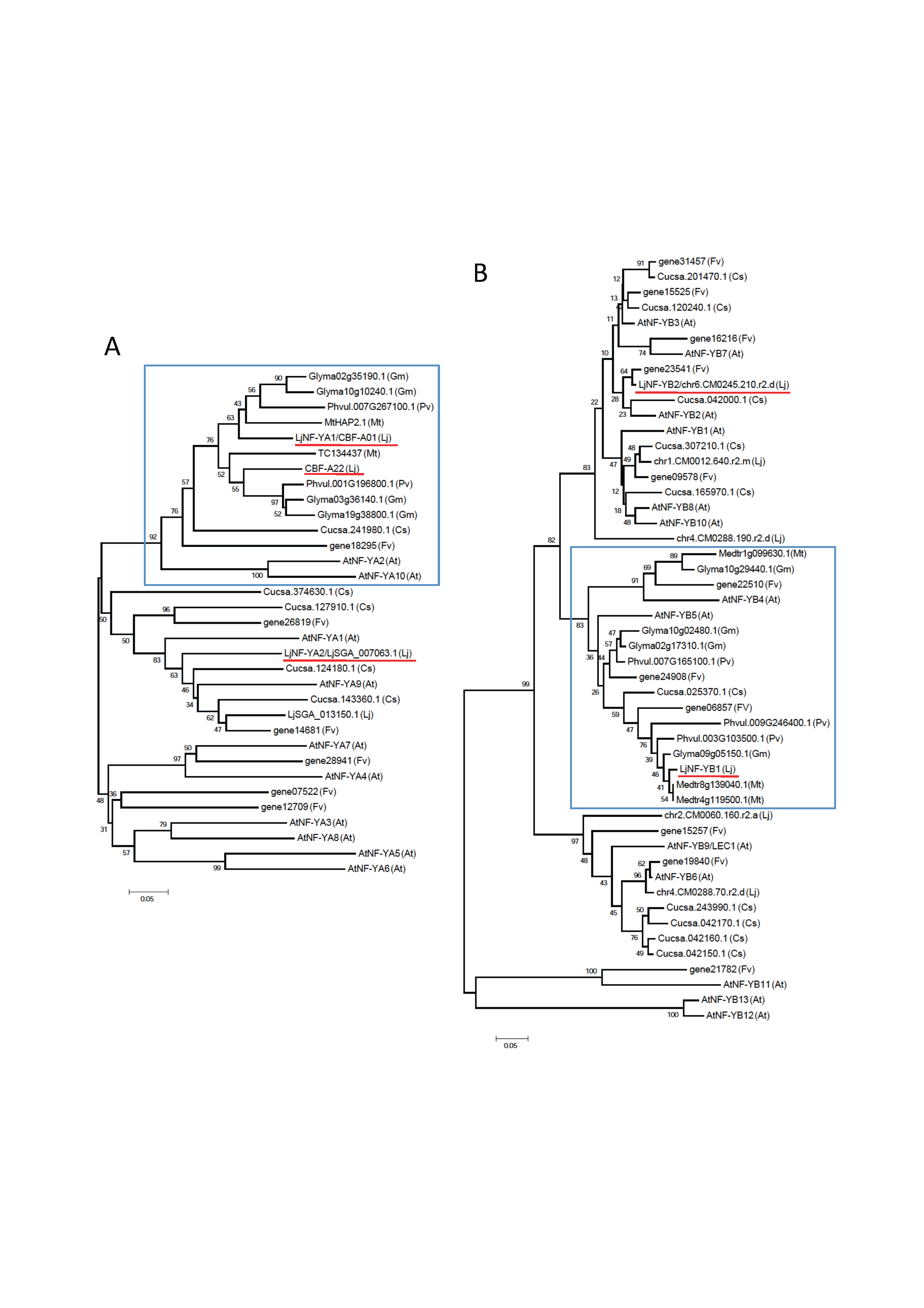

Supplement: Figure S3 — Phylogenetic trees of NF-Y subunit A and B proteins. The evolutionary histories of NF-YA (A) and NF-YB (B) proteins from non-legumes [Arabidopsis thaliana (At), Cucumis sativus (Cs), Fragaria vesca (Fv)], and legumes [Glycine max (Gm), L. japonicus (Lj), M. truncatula (Mt), and Phaseolus vulgaris (Pv)] were inferred using the Minimum Evolution method [79]. The trees are drawn to scale, with branch lengths in the same units as those of the evolutionary distances used to infer the phylogenetic tree. Evolutionary analyses were conducted in MEGA5 [80]. Clades containing LjNF-YA1 and LjNF-YB1 are indicated by blue boxes. NF-Y proteins of G. max, M. truncatula, and P. vulgaris in these clades are shown. Red underlines indicate Lotus NF-Y proteins we analyzed. (TIFF) [file pgen.1003352.s003.tif]

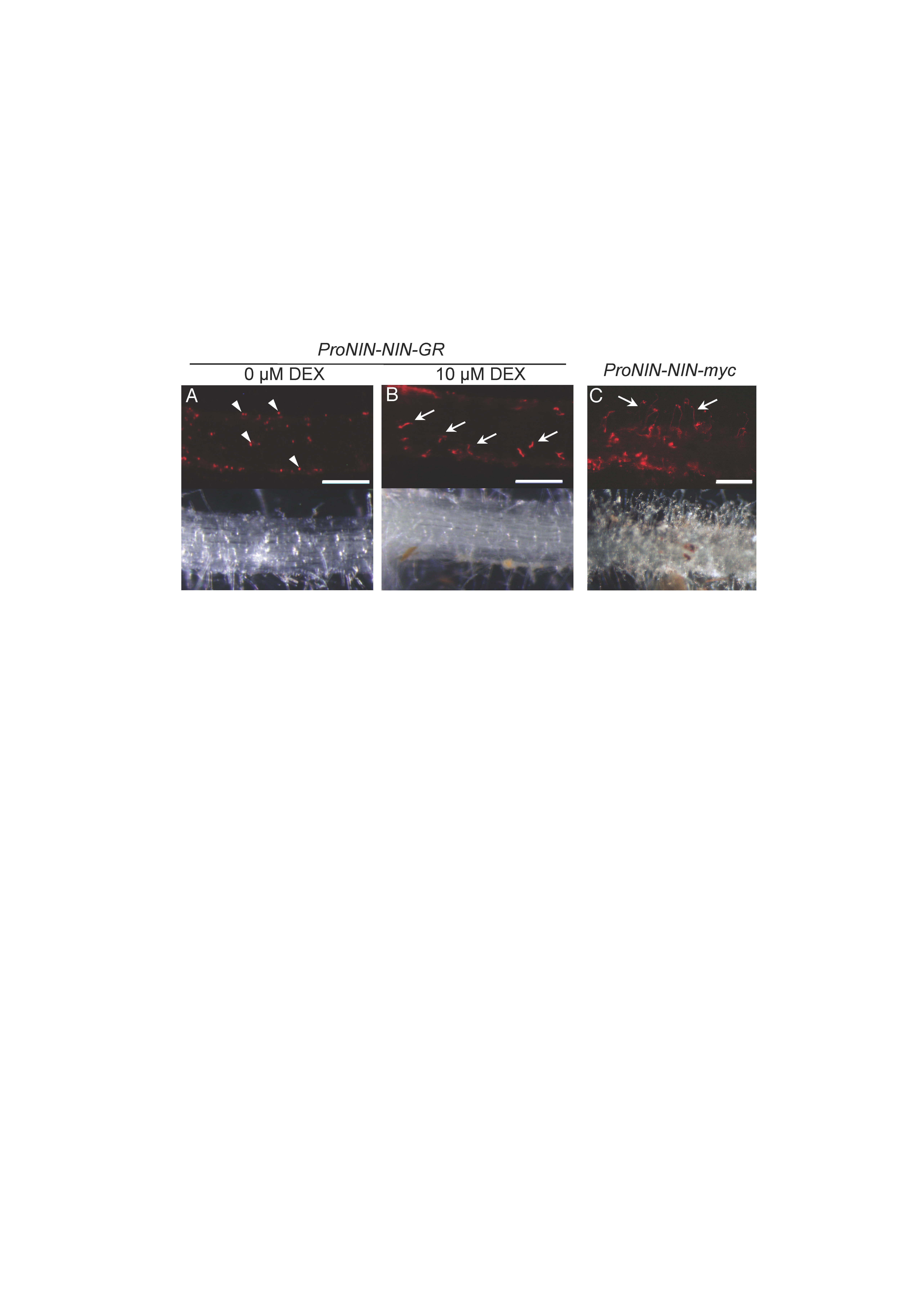

Supplement: Figure S4 — Suppression of the infection thread-defective phenotype of nin-2 mutants. nin-2 roots were transformed with either ProNIN-NIN-GR (A,B) or ProNIN-NIN-myc (C), and inoculated with DsRed-labeled M. loti for 2 weeks. ProNIN-NIN-GR rescued the infection thread-defective phenotype depending on DEX. Arrowheads and arrows indicate microcolonies and infection threads visualized by DsRed. These NIN constructs suppressed the defect in infection thread development. However, the defect in root nodule organogenesis was not rescued. Bar: 20 µm. (TIFF) [file pgen.1003352.s004.tif]

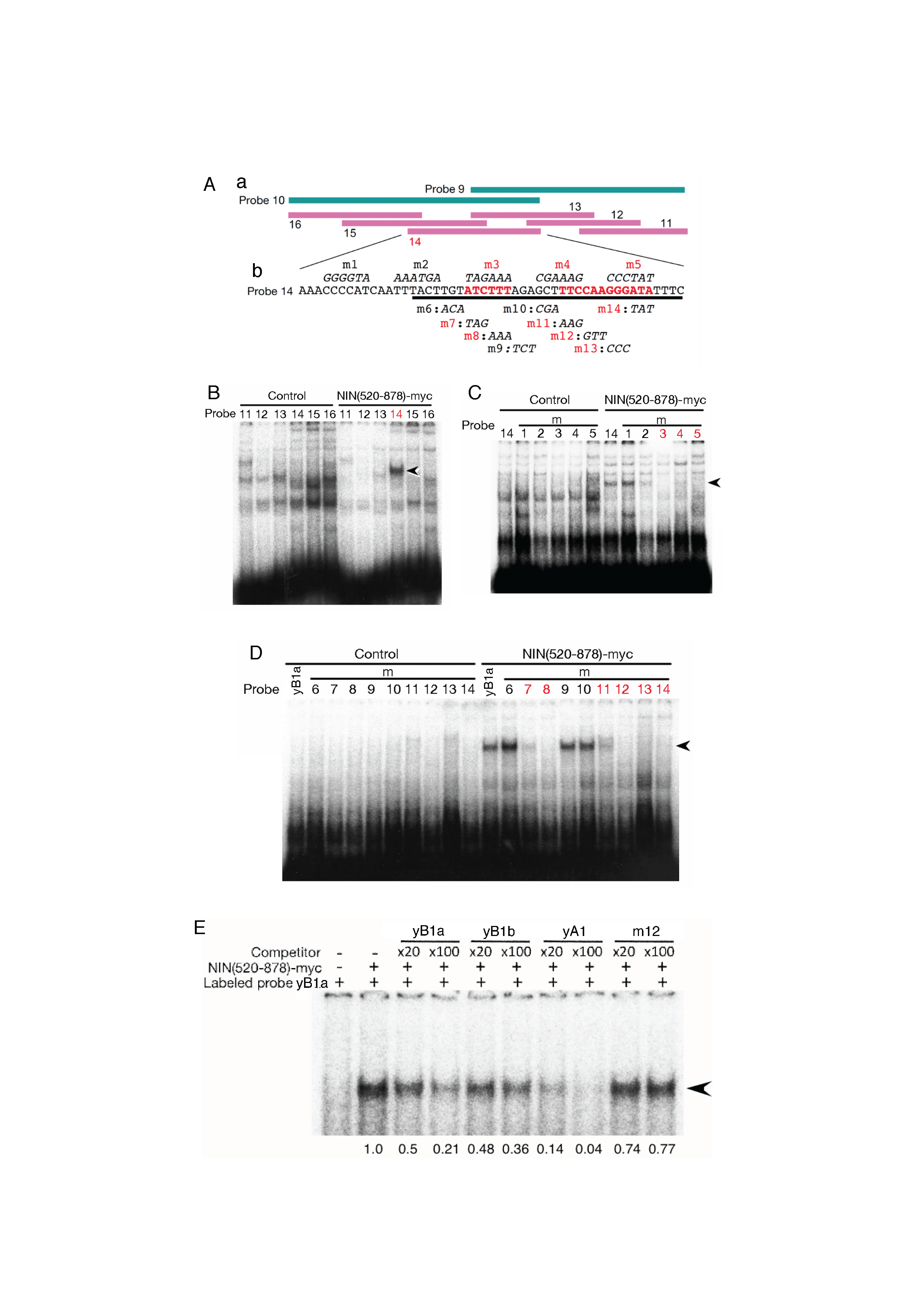

Supplement: Figure S5 — NIN binds to specific nucleotide sequences in promoter regions of NIN-target genes. (A–D) Identification of NIN-binding nucleotide sequences in the LjNF-YB1 promoter. (A) Probes used for EMSA in (B–D). (a) Green and red lines represent probes for the LjNF-YB1 promoter shown in Figure 2A (probe 9 and 10) and those used in (B), respectively. (b) A nucleotide sequence of probe 14 and nucleotide substitutions in probes m1–14 are shown. The underline indicates probe NBS-yB1a. (B–D) EMSA using NIN(520–878)-myc. Arrowheads indicate mobility-shifted probes due to binding of the NIN protein. (B) Probes shown in (Aa) were examined. (C) Probe 14 and its derivatives (m1–5) were examined. (D) Probe NBS-yB1a and its derivatives (m6–14) were examined. Note that nucleotide substitutions in m3, m5, m8, and m12–14 abolished NIN-binding, while those in m4, m7, and m11 diminished the probes' affinities to NIN. (E) Competition assay using the NIN-target nucleotide sequences that were found in the LjNF-YB1 and LjNF-YA1 promoters. The NIN protein and labeled probe NBS-yB1a were incubated with different amounts of unlabeled competitors, NBS-yB1a, -yB1b, and -yA1, and m12. Relative band intensities are shown at the bottom of lanes. (TIFF) [file pgen.1003352.s005.tif]

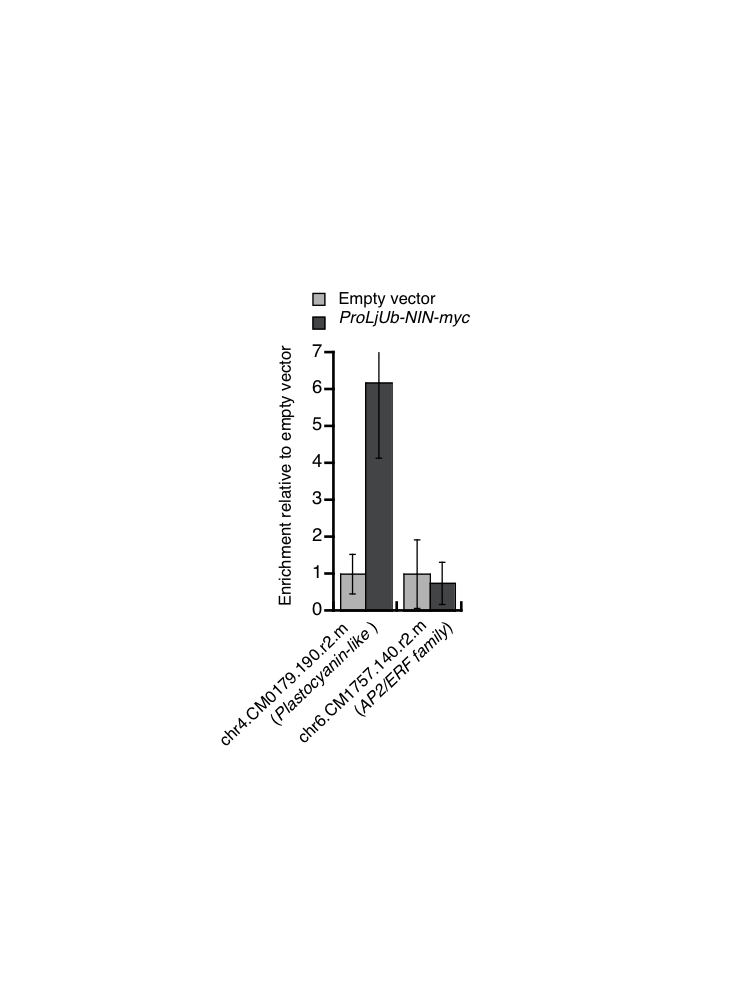

Supplement: Figure S6 — NIN binds to the promoter of chr4.CM0179.190.r2.m in vivo. ChIP analysis of chromatin suspensions that were prepared from roots transformed with either ProLjUb-NIN-myc or an empty vector. chr6.CM1757.140.r2.m is a control for RT-PCR. The levels of enrichment compared with those obtained from the empty vector are shown. Data are means and SDs from 3 biological repeats. (TIFF) [file pgen.1003352.s006.tif]

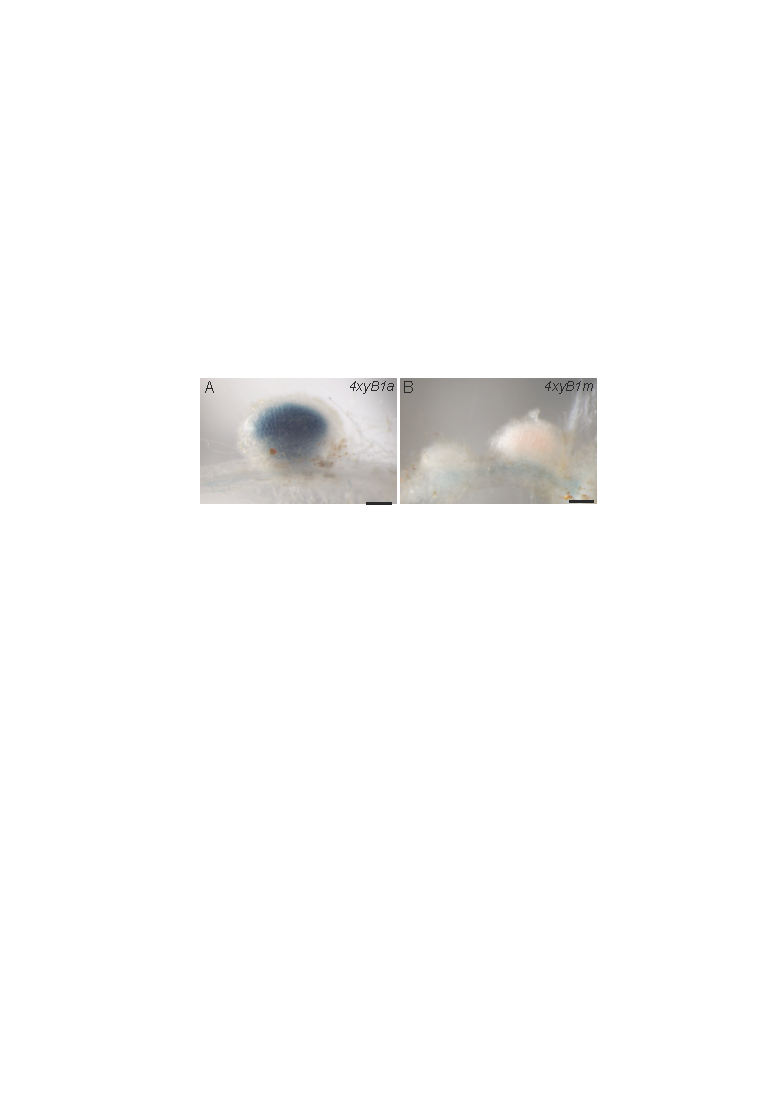

Supplement: Figure S7 — Expression of GUS in developing root nodules. Roots were transformed with either 4xyB1a-GUS (A) or 4xyB1m-GUS (B) and inoculated with M. loti. Bars: 0.2 mm (TIFF) [file pgen.1003352.s007.tif]

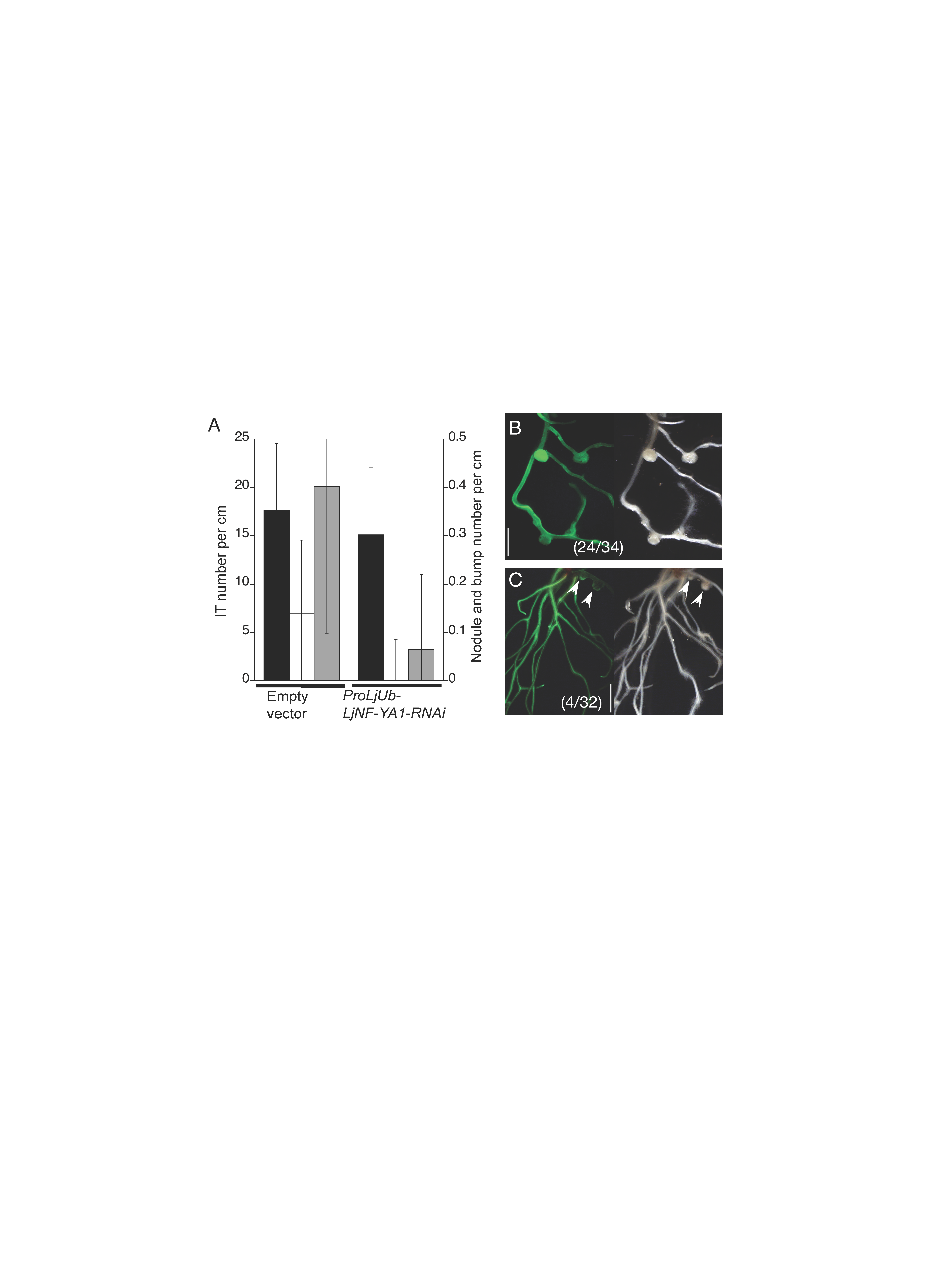

Supplement: Figure S8 — Phenotype of roots transformed with the LjNF-YA1 RNAi construct. (A) Quantitative analyses of infection threads (ITs; black bars), bumps (white), and root nodules (gray) formed on roots that were transformed with either an empty vector (n = 13) or ProLjUb-RNAi-LjNF-YA1 (n = 13). Plants were inoculated with M. loti for 14 days. (B,C) Suppression of spontaneous root nodule formation by LjNF-YA1 RNAi. Roots transformed with either the empty vector (B) or ProLjUb-RNAi-LjNF-YA1 (C) were generated from stably transformed plants carrying Pro35S-CCaMKT265D, and cultured in the absence of M. loti. Fluorescent images of GFP as a transformation marker are shown in the left panels and bright field images are shown on the right. Arrowheads indicate spontaneous root nodules formed on roots without GFP expression. The fractions of plants that formed spontaneous root nodules in roots with GFP expression are shown in parentheses. Bars: 2 mm. (TIFF) [file pgen.1003352.s008.tif]

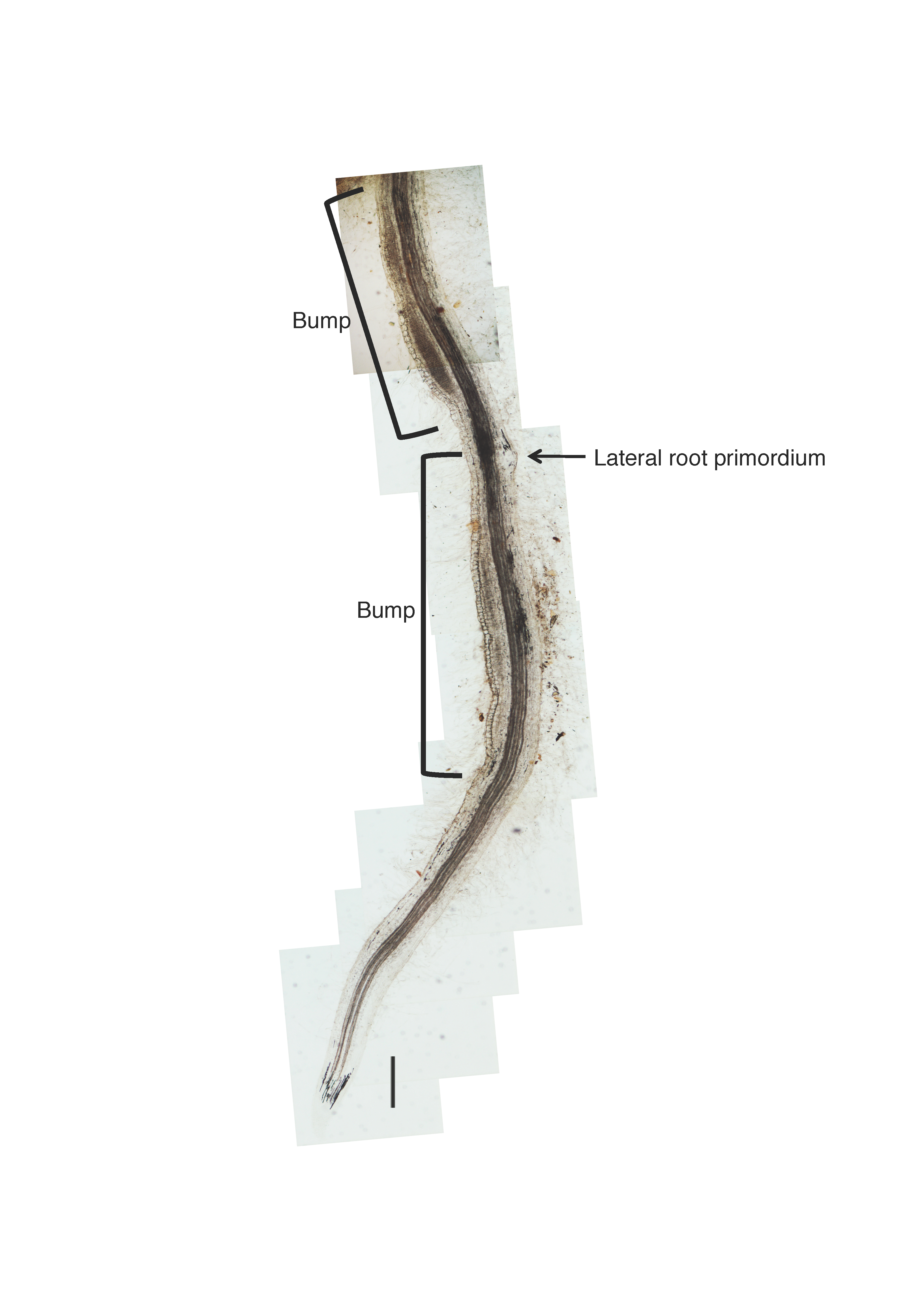

Supplement: Figure S9 — Bumps in a nin-2 root that was transformed with ProLjUb-NIN. The plant was cultured for 7 weeks after transformation in the absence of M. loti. Bar: 2 mm. (TIFF) [file pgen.1003352.s009.tif]

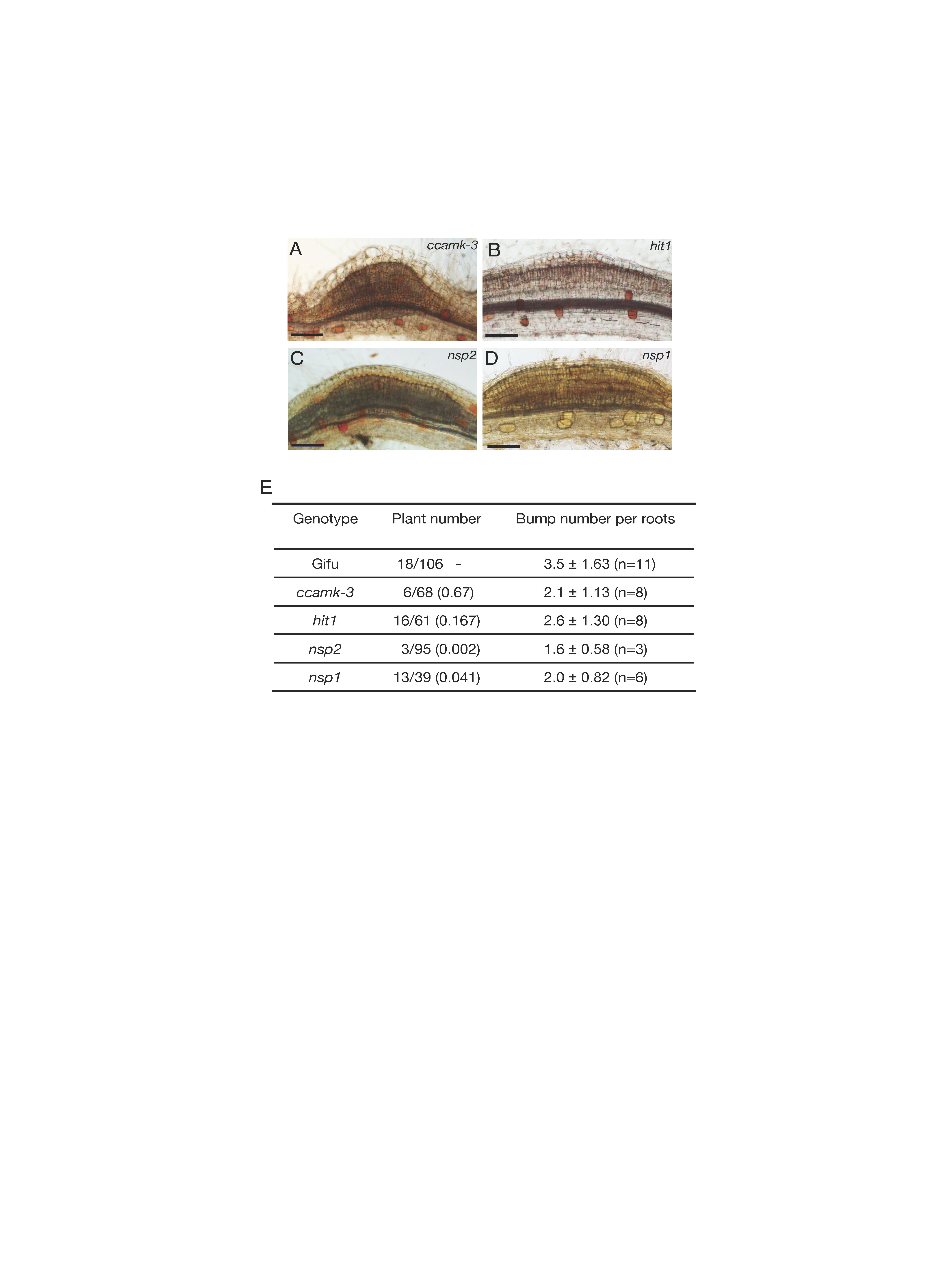

Supplement: Figure S10 — Bump formation in symbiotic mutants caused by NIN overexpression. (A–E) Bumps formed on roots of ccamk-3 (A), hit1 (B), nsp2 (C), and nsp1 (D) are shown. Roots were cultured in the absence of M. loti for 6 weeks. (E) Quantitative analysis of bump formation caused by NIN overexpression. The “Plant number” column shows the fractions of plants that formed bumps. The P-values from Fisher's exact test comparing data with those from the Gifu plants are shown in parentheses. The mean numbers of bumps per root and SDs are shown in the third column, with numbers of analyzed roots in parentheses. Bars: 0.1 mm in (A–D). (TIFF) [file pgen.1003352.s010.tif]

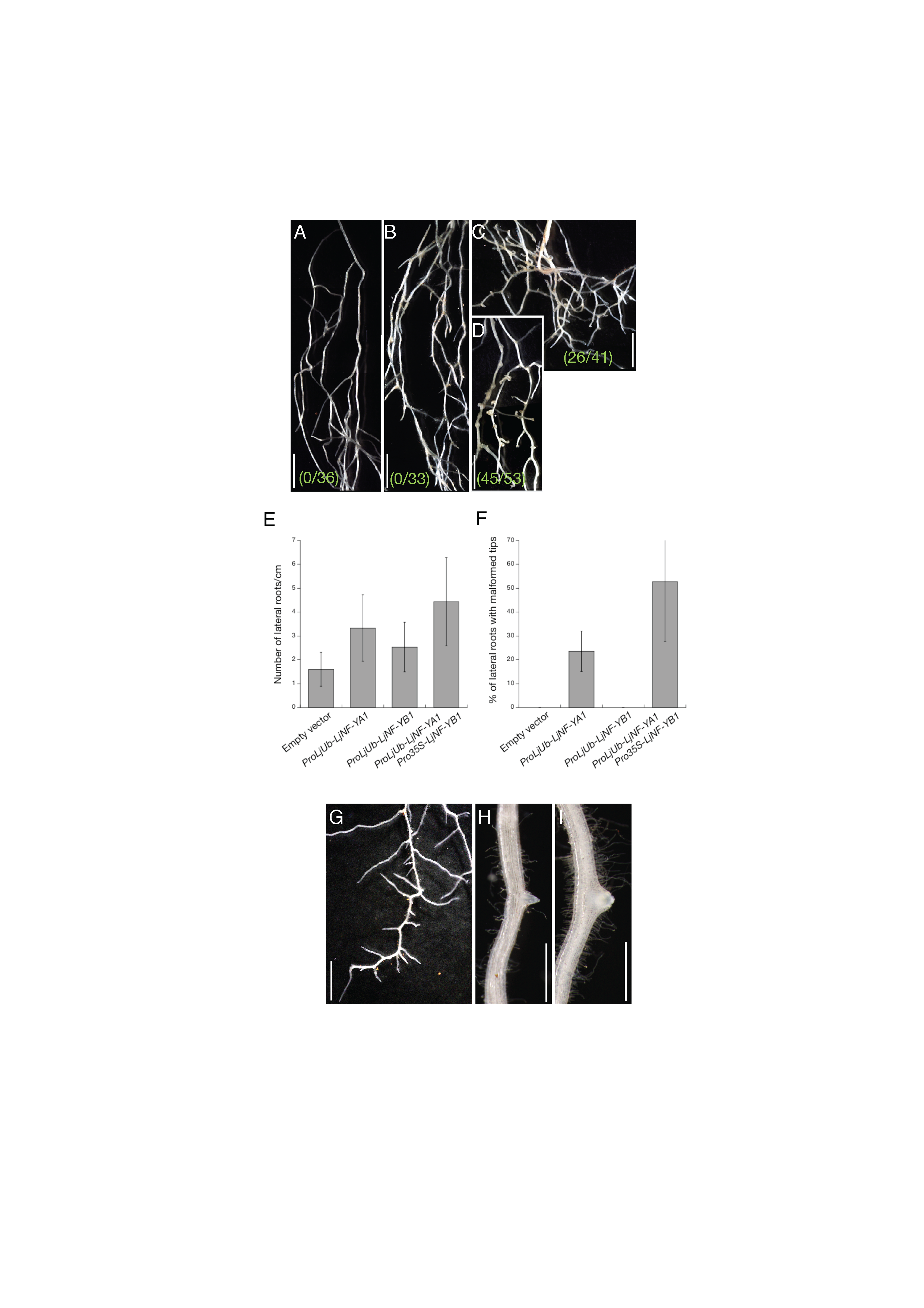

Supplement: Figure S11 — Phenotypes of roots overexpressing NF-Y subunit genes. (A–D) Roots that were transformed with either an empty vector (A), ProLjUb-LjNF-YB1 (B), ProLjUb-LjNF-YA1 (C), or ProLjUb-LjNF-YA1 Pro35S-LjNF-YB1 (D). The fractions of plants with malformed lateral roots are shown in parentheses. For (A–C), plants showing expression of a GFP selection marker were analyzed. For (D), plants that generated hairy roots were analyzed, because the selection marker was substituted by the LjNF-YB1 cDNA in the double overexpression construct. (E) Quantifications of lateral roots formed on roots that were transformed with the empty vector (n = 29), ProLjUb-LjNF-YA1 (n = 33), ProLjUb-LjNF-YB1 (n = 33), or ProLjUb-LjNF-YA1 Pro35S-LjNF-YB1 (n = 35). The means and SDs are shown. (F) Proportions of lateral roots with malformed tips in roots transformed with the empty vector (n = 8), ProLjUb-LjNF-YA1 (n = 8), ProLjUb-LjNF-YB1 (n = 6), or ProLjUb-LjNF-YA1 Pro35S-LjNF-YB1 (n = 16). The means and SDs are shown. (G–I) Roots transformed with either ProLjUb-LjNF-YA1 Pro35S-LjNF-YB2 (G,I) or the empty vector (H). Bars: 5 mm in (A–D,G); 1 mm in (H,I). (TIFF) [file pgen.1003352.s011.tif]

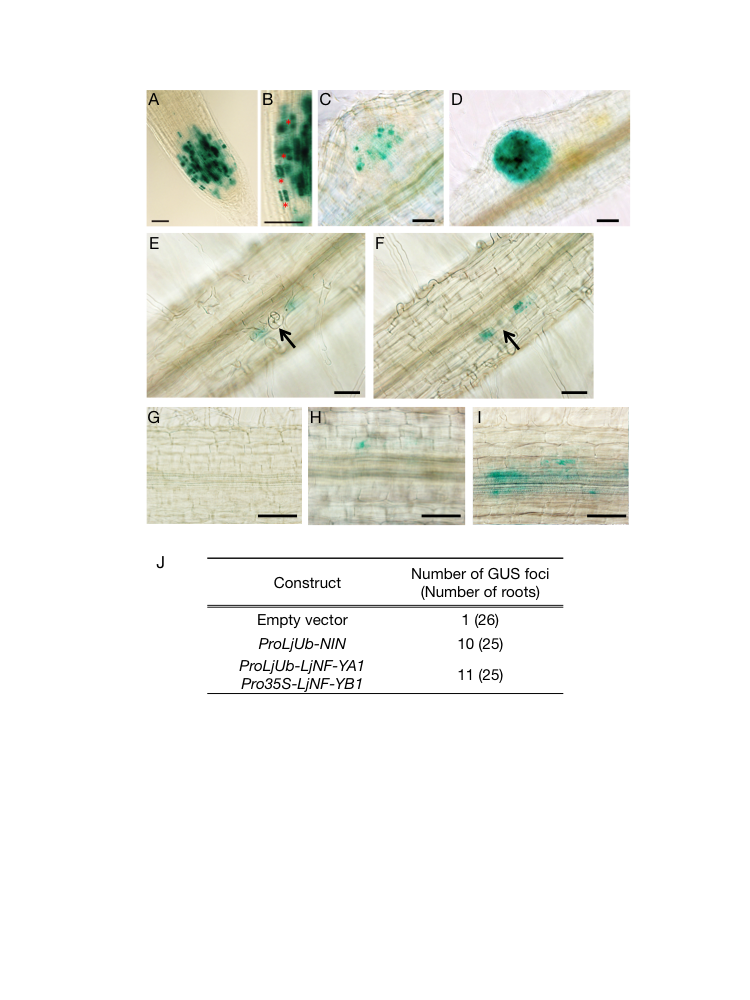

Supplement: Figure S12 — Expression of ProLjCycB1;1-CycB1;1(NT)-GUS in L. japonicus roots. (A–D) GUS staining in a primary root tip (A,B), a lateral root primordium (C), and a root nodule primordium (D) are shown. (B) Higher magnification of (A). Asterisks indicate GUS staining in adjacent daughter cells. Note that ProLjCycB1;1-CycB1;1(NT)-GUS shows typical dot-like expression patterns of Cyclin B1 as observed in Arabidopsis and soybean [81], [82]. (E,F) GUS staining in cortical cells beneath a root hair cell that was infected by M. loti. Images in (E) and (F) are the same root region focused on the epidermis (E) and the cortex (F). Arrows indicate a position of a curled root hair. (G–I) ProLjCycB1;1-CycB1;1(NT)-GUS expression in roots that were transformed with either an empty vector (G), ProLjUb-NIN (H), or ProLjUb-LjNF-YA1 Pro35S-LjNF-YB1 (I). Roots were cultured on agar media for 16 days in the absence of M. loti. (J) The number of GUS foci in cortical layers of roots that were transformed with indicated constructs. Bars: 50 µm in (A–F), 100 µm in (G–I). (TIFF) [file pgen.1003352.s012.tif]
