# Supplementary material for: NODULE INCEPTION Directly Targets NF-Y Subunit Genes to Regulate Essential Processes of Root Nodule Development in Lotus japonicus
Source: PLoS Genet. 2013 Mar 21;9(3):e1003352. doi: 10.1371/journal.pgen.1003352 (PMC3605141; doi:10.1371/journal.pgen.1003352)
Supplement: Table S1 — Sequences of oligo-nucleotides used for primers and EMSA probes. Primer and probe sequences used in the experiments are shown. Construction of plasmids (A), Gene expression analysis (B), ChIP analysis and EMSA (C). (PDF) [file pgen.1003352.s013.pdf]

Table S1. Sequences of oligo-nucleotides used for primers and EMSA probes.

A <u>Primers for construction of plasmids</u>

Primers for *LjNF-YA1* promoter

5’ AAGGATCCATATAAGTTTGTAAAAGCGTCACTAATTT 3’

5’ AAGCGGCCGCGTCTAGGGATTAGGCTCCAAAATG 3’

Primers for *LjNF-YB1* promoter

5’ AAGGATCCATCATCCAATCACATTTGAGATAAGTG 3’

5’ AAGCGGCCGCTCTCTTGATGTCCCAGGAATAAATTAAC 3’

Primers for *LjNF-YA1* cDNA

5’ ACGTCGACATGGCGATGCAAACTGTTTATCTGAA 3’

5’ AAGCGGCCGCTCAGACTTTAAGGTTGAAGCAGCTT 3’

Primers for *LjNF-YB1* cDNA

5’ ACGTCGACATGGAGGATCACATTGGAAGCAG 3’

5’ AAGCGGCCGCTCAGTGCCTAGTTCCTCTTTGG 3’

Primers for *RNAi-LjNF-YA1*

5’ TCGGAAAGGACACAAGTGGGATTG 3’

5’ CACCCAATGTCCCCAATGCCCTGGCAGG 3’

Primer for *RNAi-LjNF-YB1*

5’ TGCAGATCCAATGAGAAGGTATTTG 3’

5’ CACCTTAAACTGGCTTTAAGATGGAGA 3’

Primers for *GFP*

5’ AACTCGAGGATGGTGAGCAAGGGCGAGG 3’

5’ AAACTCGAGGTTATAACATGCCGGGCTTGTACAGC 3’

Primers for *Gal4DBD*

5’ AAGCGGCCGCGGATGAAGCTACTGTCTTCTATCGAACAA 3’

5’ AACTCGAGTCACATATGAGTCAAAGCTGGGTATTG 3’

Primers for 4xUAS with CaMV35 minimal promoter

5’ CCGGTCGACTCTAGAGGATCC 3’

5’ AAGCGGCCGCTCAGCGTGTCCTCTCCAAATGA 3’

Primers for CaMV35 minimal promoter

5’ AACTCGAGTCAGCGTGTCCTCTCCAAATGA 3’

5’ AAGCGGCCGCTCGACCTCGATCGAGATCTTCG 3’

Primers for synthesizing 4xyBa

5’ TTGTCGACATCTTTAGAGCTTTCCAAGGGATATTTCGCGGATCTTTAG-AGCT 3’

5’ CGCGGATCTTTAGAGCTTTCCAAGGGATATTTCTTGTATCTTTAGAGC-

TTTCCAAGGGATATTTCCGCCA 3’

5’ AAGGTTATCGGACCGAAATATCCCTTGGAAAGCTCTAAAGATGGCG-GAAATATCCCTTG 3’

Primers for synthesizing 4xyBam

5’ TTGTCGACATCAAAAGAGCTTTCGTTGGGATATTTCGCGGATCAAAA-GAGCT 3’

5’ CGCGGATCAAAAGAGCTTTCGTTGGGATATTTCTTGTATCAAAAGAG-

CTTTCGTTGGGATATTTCCGCCA 3’

5’ AAGGTTATCGGACCGAAATATCCCAACGAAAGCTCTTTTGATGGCGG-AAATATCCCAAC 3’

Primers for *LjCycB1;1*

5’ CTGGTACCCATGGGTTTGGG 3’
5’ TCCCCCGGGCTTGCTTCGAGCAGTGAGCAC 3’

B Primers for gene expression analysis

For *polyubiqutin*

5’ ATGCAGATCTTTTGTGAAGAC 3’

5’ ACCACCACGGAAGACGGAG 3’

For *NIN*

5’ AACTCACTGGAAACAGGTGCTTTC 3’

5’ CTATTGCGGAATGTATTAGCTAGA 3’

For *LjNF-YA1*

5’ GAAGCTGCTTCAACCTTAAAGTC 3’

5’ CGAGATGTAGAACTGAACTTGTCAC 3’

For *LjNF-YB1*

5’ GAGAATGGAGGATCACATTGGAAG 3’

5’ GGTAGAATCTGCTTCATGATCCTG 3’

For chr4.CM0046.1620.r2.m

5’ CACTTCCTGGTATTGCACTTGTC 3’

5’ CCTGGGCTGATACATAAGCTGGT 3’

For chr2.CM0008.630.r2.m

5’ GTTGGGTGTTATATGTTCCCAAGG 3’

5’ CTCCTGACGATAAGAATGATCAAGG 3’

For chr4.CM0179.190.r2.m

5’ TTCACAAGATTCACTCAGCCAGTG 3’

5’ CCTTCCAAGTATACCTCATGGTC 3’

For chr6.CM1757.140.r2.m

5’ ACTTCCAACATTGGAAGCTCCTC 3’

5’ GCCTGAGTTAGAAGAATCATTGGAG 3’

For chr3.CM0996.230.r2.a

5’ GTTCAACAACGCCTAAGTCCAAAG 3’

5’ AGTCCTTGACTGGTACTCTTTGCA 3’

For chr5.CM0311.320.r2.m

5’ CTCCTCCACCAGGCCAAAATTAC 3’

5’ GGTCAATTGGGATAGGAATGTTTGG 3’

For chr2.CM0081.540.r2.d

5’ CGGTGACGGTAATGGTGACAGA 3’

5’ CAGCCAACACTGCAAAAGCTTTC 3’

For CBF-A22

5’ GGTAACAGCTGCAACCTTAACACT 3’

5’ GGATGGATTGCCTTAAGACCAGA 3’

For LjSGA_007063.1

5’ ACTAAAGAATCACAACCTACTGGAAG 3’

5’ GATTTGAAGCACAAGCCTGTGGA 3’

C **Primers for ChIP analysis and probes for EMSA**

Primers for ChIP analysis of *LjNF-YA1* promoter region 1

5’ CGTAGAGACTAACTTGACCGACTT 3’

5’ TACGGACAAAATAGAGTGAATGGTGAA 3’

Primers for ChIP analysis of *LjNF-YA1* promoter region 2 and for EMSA probe 6

5’ CCTAGTAAATATAAGGCAATCACTTAGG 3’

5’ GTTACTCCAGATCAGGTGGTTCA 3’

Primers for ChIP analysis of *LjNF-YA1* promoter region 3 and for EMSA probe 9

5’ GAGAAAATGCTAGTTTTTAAAAAGAGAACCT 3’

5’ CAGGAAAATGCCTGTCCAGATAAG 3’

Primers for ChIP analysis of *LjNF-YA1* promoter region 4

5’ CTCGAGTGGTAAAAATTGGTGGAC 3’

5’ AGGATTGAACTCCCAGATGTTCAAATA 3’

Primers for ChIP analysis of *LjNF-YA1* promoter region 5

5’ GACTACTTAAGCTAAACTGAATTGAAGTC 3’

5’ GTGTAGTGTATCATTAGGATACCTCTA 3’

Primers for EMSA probe 7 of *LjNF-YA1* promoter

5’ TGGTTAATCACAGGAAAAATGCTGTTC 3’

5’ CCTAAGTGATTGCCTTATATTTACTAGG 3’

Primers for EMSA probe 8 of *LjNF-YA1* promoter

5’ CTTATCTGGACAGGCATTTTCCTG 3’

5’ GAACAGCATTTTTCCTGTGATTAACCA 3’

Primers for EMSA probe 10 of *LjNF-YA1* promoter

5’ GTGATCAAAATAACAAATAAGAATTGAAATAACTC 3’

5’ AGGTTCTCTTTTTAAAAACTAGCATTTTCTC 3’

Primers for ChIP analysis of *LjNF-YB1* promoter region 1

5’ ACACCCAAAATCTTCTCTCCATAATTG 3’

5’ CTCACATCTACGTGTAGTGCATG 3’

Primers for ChIP analysis of *LjNF-YB1* promoter region 2 and for EMSA probe 5

5’ CTGCAGAATCAGAACCCATGATTC 3’

5’ CTAAATATGCACGGCTACTAAATTTATC 3’

5’ primer for ChIP analysis of *LjNF-YB1* promoter region 3 and for EMSA probe 10 and 16

5’ TCACTTTGGTGGATGTGGATATACAA 3’

3’ primer for ChIP analysis of *LjNF-YB1* promoter region 3

5’ ATGGATGCTGAGAAGGCCTTTGA 3’

Primers for ChIP analysis of *LjNF-YB1* promoter region 4

5’ CTAATATGAAATGCCATATATCACTTC 3’

5’ GTTTTGAGTGTGAAGGTTATATAATTGCAT 3’

5’ primer for EMSA probe 6 of *LjNF-YB1* promoter

5’ CTTGGCACCATTCGCATGTCAC 3’

3’ primer for EMSA probe 6 and 14 of *LjNF-YB1* promoter

5’ TCATCTATACAGAGCTTAGTCACATATGAATGAT 3’

Primers for EMSA probe 7 of *LjNF-YB1* promoter

5’ AATGACTCTGCACTCCAATTGATTGA 3’

5’ GTGACATGCGAATGGTGCCAAG 3’

Primers for EMSA probe 8 of *LjNF-YB1* promoter

5’ ACTTGTATCTTTAGAGCTTTCCAAGG 3’

5’ GAATCATGGGTTCTGATTCTGCAG 3’

5’ primer for EMSA probe 9 and 13 of *LjNF-YB1* promoter

5’ CTTTAGAGCTTTCCAAGGGATATTTC 3’

3’ primer for EMSA probe 9 and 11 of *LjNF-YB1* promoter

5’ GTTAATGGATGCTGAGAAGGCCT 3’

3’ primer for EMSA probe 10 of *LjNF-YB1* promoter
5’ GAAATATCCCTTGGAAAGCTCTAAAG 3’

5’ primer for EMSA probe 11 of *LjNF-YB1* promoter
5’ TCTTTTGAGCGGCCTCAAAGGCC 3’

Primers for EMSA probe 12 of *LjNF-YB1* promoter
5’ ATTTCATATTCATCTTCACTCTTT 3’
5’ GGCCTTTGAGGCCGCTCAAAAGA 3’

3’ primer for EMSA probe 13 of *LjNF-YB1* promoter
5’ AAAGAGTGAAGATGAATATGAAAT 3’

5’ primer for EMSA probe 14 of *LjNF-YB1* promoter
5’ AAACCCCATCAATTTACTTGTATCTTTAG 3’

Primers for EMSA probe 15 of *LjNF-YB1* promoter
5’ TACAATTCTTTACTTTCAATACAAAAAACC 3’
5’ CTAAAGATACAAGTAAATTGATGGGGTTT 3’

3’ primer for EMSA probe 16 of *LjNF-YB1* promoter
5’ GGTTTTTTGTATTGAAAGTAAAGAATTGTA 3’

Probe yA
5’ AGTTAGATTGCTTGGCCCCCTTCTTATCTGGACAGGCATTTTCCTG 3’
5’ ACAGGAAAATGCCTGTCCAGATAAGAAGGGGGCCAAGCAATCTAAC 3’

Probe yBa
5’ TACTTGTATCTTTAGAGCTTTCCAAGGGATATTTC 3’
5’ AGAAATATCCCTTGGAAAGCTCTAAAGATACAAGT 3’

Probe yBb
5’ TCTTCACTCTTTTGAGCGGCCTCAAAGGCCTTCTCAG 3’
5’ GCTGAGAAGGCCTTTGAGGCCGCTCAAAAGAGTGAAG 3’

Probe E16a
5’ ACGAAAAAACCCTTCAACCCCTCAAAAAATGAACAG 3’

5’ CCTGTTCATTTTTTGAGGGGTTGAAGGGTTTTTTCG 3’

Probe E16b

5’ AATTACATAATCCTTAAAGATAACGAAAAGGTGAATT 3’

5’ AAATTCACCTTTTCGTTATCTTTAAGGATTATGTAAT 3’
